# Machine learning-driven discovery of NETs-associated diagnostic biomarkers and molecular subtypes in tuberculosis

**DOI:** 10.3389/fcimb.2025.1591464

**Published:** 2025-10-01

**Authors:** Shoupeng Ding, Yimei Yang, Chunxiao Huang, Yuyang Zhou, Zihan Cai

**Affiliations:** ^1^ Department of Laboratory Medicine, Gutian County Hospital, Ningde, China; ^2^ Department of Microbiology and Immunology, School of Basic Medical Sciences, Dali University, Dali, China; ^3^ Department of Medical Laboratory, Siyang Hospital, Suqian, China; ^4^ Oncology and Laboratory Immunology Research Innovation Center, Siyang Hospital, Suqian, China

**Keywords:** tuberculosis, machine learning, neutrophil extracellular traps, molecular subtypes, mycobacterium tuberculosis

## Abstract

**Object:**

NETs constitute a pivotal mechanism in the pathogenesis and progression of TB. Despite their recognized importance, the genetic underpinnings of NETs in TB remain inadequately elucidated. Accordingly, the present study endeavors to delineate the molecular characteristics of NRGs in TB, with the objective of reliably identifying associated molecular clusters and biomarkers.

**Methods:**

Gene expression profiles were analyzed from integrated datasets retrieved from the GEO database. Differential analysis, WGCNA, and an ensemble of 113 machine learning algorithms were employed to identify the core NETs genes. Subsequently, TB patients were stratified into distinct subtypes based on the expression profiles of these core genes, and the differences in immune infiltration characteristics among the subtypes were systematically compared. Finally, RT-qPCR was utilized to validate the differential expression of the key NETs core genes.

**Results:**

Analysis of the integrated GSE83456 and GSE54992 datasets yielded 630 DEGs. WGCNA subsequently identified a module comprising 1,252 genes, from which 26 key NETs genes were extracted via intersection with known NRGs. Among the ensemble of 113 machine learning methods, the “StepgIm[both]+RF” algorithm demonstrated superior performance, ultimately identifying six core NETs genes. Consensus clustering based on the expression profiles of these core genes stratified patients into two distinct subtypes. Functional enrichment analysis further underscored the predominance of immune-related pathways in subtype B. Moreover, immune infiltration analysis revealed marked differences in immune cell composition between the subtypes, thereby confirming a close association between the core NETs genes and these immunological disparities.

**Conclusion:**

Core NETs genes are pivotal in the pathogenesis and progression of tuberculosis, and they hold significant promise as novel biomarkers for the early diagnosis and targeted treatment of TB.

## Introduction

Tuberculosis (TB) is a highly contagious chronic disease caused by *Mycobacterium tuberculosis* (MTB) and remains one of the foremost infectious diseases responsible for a substantial number of deaths globally. At present, tuberculosis is the second leading cause of infectious disease-related mortality, surpassed only by COVID-19, and is responsible for approximately 1.3 million deaths each year (Bagcchi, 2023; Ding et al., 2024b). Although the Bacillus Calmette-Guérin (BCG) vaccine—derived from an attenuated strain of bovine *Mycobacterium*—effectively diminishes the risk of severe TB manifestations, including meningitis and disseminated tuberculosis in children, its protective efficacy against adult TB remains limited (Singh et al., 2022).

Regarding treatment, isoniazid—a first-line anti-TB medication—is widely employed for its potent bactericidal properties. Nonetheless, its clinical application is frequently complicated by hepatotoxicity, as studies have indicated that between 5% and 33% of patients experience drug-induced liver damage during treatment (Ding et al., 2024a; Jiang et al., 2024). Such hepatotoxicity not only markedly diminishes the efficacy and cure rates of TB treatment but also substantially escalates the mortality risk among patients. Therefore, an in-depth exploration of the pathogenesis and progression of TB is imperative to develop safer and more efficacious alternative therapeutic strategies, rendering it an urgent priority in contemporary research.

The pathogenesis of TB is exceedingly complex, encompassing a diverse array of immune cells—including macrophages, lymphocytes, and neutrophils—and characterized by perturbations in both innate and adaptive immune responses (Kanabalan et al., 2021; Cavalcante-Silva et al., 2023). Neutrophils, the most prevalent innate immune cells in the human body, have traditionally been regarded as the primary line of defense against pathogenic invasion (Li et al., 2023). Nevertheless, the advent and meticulous investigation of neutrophil extracellular traps (NETs) have revolutionized our understanding of neutrophil functionality and their multifaceted role in immune defense mechanisms. NETs are intricate, web-like structures composed of chromatin and antimicrobial proteins secreted by neutrophils (Shen and Lin, 2024), initially presumed to serve predominantly in pathogen sequestration and neutralization. Recent investigations, however, have elucidated that NETs play a critical role in both the initiation and progression of TB. Dang et al. (Dang et al., 2018) demonstrated that MTB induces NET release via its extracellular sphingomyelinase Rv0888 and concurrently activates myeloperoxidase (MPO), thereby exacerbating pulmonary lesions through caspase-3-mediated apoptotic pathways. Moreover, NETs induced by MTB further stimulate macrophages to secrete pro-inflammatory cytokines, including IL-6 and TNF-α, thereby recruiting additional neutrophils to the infection locus—a process that intensifies inflammation and exacerbates pulmonary damage (Braian et al., 2013). Beyond inflicting tissue damage, excessive NET release may impair the antimicrobial capacity of neutrophils and foster a nutrient-rich necrotic microenvironment that promotes MTB proliferation, thereby accelerating the pathological progression of TB (Francis et al., 2014). Notably, NET-associated biomarkers exhibit significant potential for diagnostic and staging applications in TB. Meier et al. (Meier et al., 2022) observed that circulating NET levels in patients who eventually progressed to TB were markedly elevated as early as six months prior to clinical diagnosis. Similarly, Melo et al. (de Melo et al., 2018) reported a pronounced increase in the levels of citrullinated histone H3—a well-recognized NET-related biomarker—in the peripheral blood of TB patients. Consequently, a comprehensive elucidation of the mechanistic underpinnings of NETs in TB, coupled with an exploration of the dynamic fluctuations of NET levels in patient blood, not only facilitates the early prediction of TB progression but also furnishes critical evidence for the development of innovative therapeutic targets. Nevertheless, systematic investigations into neutrophil-related genes (NRGs) in TB remain scant and warrant further scholarly exploration.

**Figure 1:**
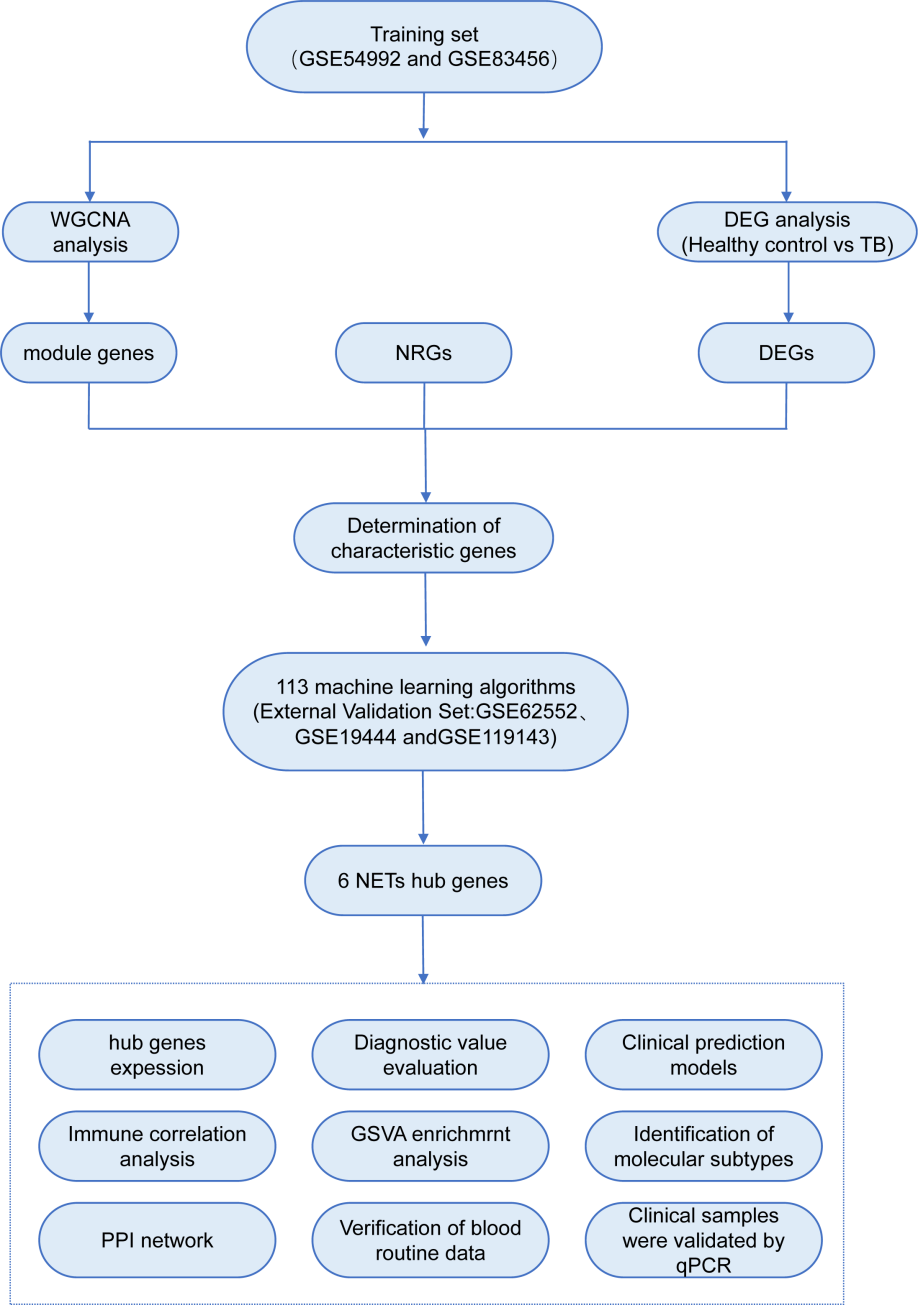
Experimental flowchart.

In recent years, the advent and widespread application of gene chip technology have rendered bioinformatics indispensable in the assessment of disease onset, progression, and the identification of diagnostic and prognostic biomarkers. However, conventional differential gene expression analysis methods may inadvertently overlook pivotal biological information, and the reliability of single-chip data is frequently compromised by inter-sample variability and divergent experimental conditions. Therefore, the present study employs a multi-chip, integrative bioinformatics analysis approach to systematically investigate the transcriptional alterations of NRGs in the blood of TB patients, incorporating advanced machine learning algorithms to identify core genes. Furthermore, TB patients are stratified based on the expression profiles of these core genes, thereby elucidating the potential biological characteristics of distinct subtypes. Moreover, through experimental validation of the expression levels of these core genes in clinical samples, this study aims to furnish more reliable and comprehensive scientific evidence to support the early diagnosis and precision treatment of TB. The analytical workflow employed in this study is illustrated in **Figure 1**.

## Materials and methods

### Study population

This investigation leveraged six Gene Expression Omnibus (GEO) datasets (GSE54992, GSE83456, GSE19444, GSE34608, GSE6252, GSE119143), encompassing patients with tuberculosis (TB) and healthy controls, alongside whole blood samples from five TB patients and five healthy volunteers for RT-qPCR validation. Clinical characteristics of TB patients, which guided patient stratification and analysis, included sputum smear status, chest radiography findings (e.g., presence of cavitary lesions), and biomarkers such as C-reactive protein (CRP) concentrations and erythrocyte sedimentation rate (ESR). These attributes were chosen due to their well-documented significance in TB diagnosis and progression, as well as their established utility in clinical settings for evaluating disease severity and therapeutic response.

### Data acquisition

Six datasets (GSE54992, GSE83456, GSE19444, GSE34608, GSE62525, and GSE119143), encompassing gene expression profiles from patients with tuberculosis (TB) and healthy controls, were retrieved from the Gene Expression Omnibus (GEO) repository. Gene expression matrices were constructed using platform-specific annotation files (e.g., GPL570 for GSE54992 and GSE83456). To ensure data integrity, preprocessing steps were executed in R (version 4.4.1). Low-quality probes were excluded based on signal intensity thresholds (below the 2.5th percentile). Data were normalized using the robust multi-array average (RMA) method from the affy package to minimize variance across samples. Batch effects were ameliorated using the ComBat function from the sva package (version 3.40.0), integrating GSE54992 and GSE83456 into a cohesive training dataset, while the remaining four datasets (GSE19444, GSE34608, GSE62525, GSE119143) served as validation cohorts (**Table 1**). To identify genes associated with neutrophil extracellular traps (NETs; hereafter NRGs), we systematically interrogated multiple repositories, including GeneCards (Rebhan et al., 1997), OMIM (Mckusick, 2007), NCBI Gene (Edgar et al., 2002), and pertinent literature (Sheng and Cui, 2024), yielding a curated catalog of 403 NRGs. These NRGs were refined for relevance to TB pathology based on functional annotations and prior research.

**Table 1:** Microarray information of gene expression dataset.

Type	Dataset	Source	Sample		Platform	Group
			Control	TB		
GEO	GSE83456	PBMC	61	45	GPL10558	Training section
	GSE54992	PBMC	6	9	GPL570	
	GSE62552	PBMC	7	7	GPL16951	Validation section
	GSE19444	PBMC	12	21	GPL6947	
	GSE34608	PBMC	18	8	GPL6480	
	GSE119143	PBMC	5	7	GPL17586	

### Differential gene expression selection

Differential expression analysis between patients with tuberculosis (TB) and healthy controls was conducted using the “limma” package in R. Genes were deemed differentially expressed if they satisfied the criteria of |log2 fold change| > 0.585 (corresponding to a fold change of approximately 1.5 on a linear scale, as 2^0.585 ≈ 1.5) and an adjusted p-value (FDR) < 0.05. The log2 fold change threshold of 0.585 was selected based on the following rationale: (1) a fold change of 1.5 is widely acknowledged in gene expression studies as a biologically significant threshold, balancing sensitivity and specificity for identifying differentially expressed genes (DEGs) pertinent to tuberculosis pathology (Maertzdorf et al., 2012); (2) this threshold, coupled with an FDR < 0.05, ensures statistical rigor while capturing genes exhibiting moderate yet consistent expression changes; and (3) exploratory analyses of our dataset corroborated that a |log2 fold change| > 0.585 effectively delineated genes with robust differential expression patterns across replicates, aligning with the study’s biological hypotheses. Volcano plots and heatmaps were generated using the “ggplot2” and “pheatmap” packages to visualize the DEGs.

### Weighted gene co-expression network analysis

A weighted gene co-expression network was constructed with the “WGCNA” package to identify functional modules in TB samples. Genes with an average expression > 0.5 were selected, and outlier samples were removed using hierarchical clustering (“Hclust”). An optimal soft threshold (β, 1–20) was determined to convert the adjacency matrix into a Topological Overlap Matrix (TOM). Modules containing at least 60 genes were delineated via hierarchical clustering and subsequently consolidated. Key modules correlated with clinical features were identified, and intersecting genes between these modules and NRGs yielded critical NETs genes.

### Enrichment analysis of key NETs genes

GO and KEGG enrichment analyses were performed on the identified DEGs to elucidate the functional roles of key NETs genes. The GO analysis examined biological processes, molecular functions, and cellular components, while the KEGG analysis provided an overview of metabolic and cellular pathways, systemic functions, and disease associations (Kanehisa et al., 2023). Both analyses were executed using “clusterProfiler” and “enrichplot” in R, with significance thresholds of p < 0.05 and q < 0.05.

### Selection of key NETs genes

To select key NETs genes, we employed a combination of 12 machine learning algorithms, including Elastic Net (Enet), Ridge, Stepglm, LASSO, SVM, LDA, plsRglm, RF, GBM, XGBoost, and Naïve Bayes. Consequently, 113 combined models were constructed, and cross-validation techniques were applied to mitigate overfitting risk: one set of algorithms was used for variable selection, while another set built classification prediction models. Furthermore, the number of validation sets was appropriately augmented to ensure the robustness of the model. Model performance was evaluated across various cohorts by calculating the Area Under the Curve (AUC) of the ROC curve, and the results were visualized through heatmaps. The most optimal model from the 113 algorithms was selected for further assessment of the diagnostic sensitivity and specificity of TB.

### Expression and diagnostic value of key NETs genes

Based on the results from the most accurate algorithm, volcano plots were generated using the “ggplot2” package in R to display the expression distribution of key NETs genes. Gene functional enrichment analysis was performed using the online tool GeneMANIA (https://genemania.org/) (Warde-Farley et al., 2010), and interactions with scores exceeding 0.4 were regarded as significantly relevant. Subsequently, box plots were employed to display the expression differences of key NETs genes between TB patients and healthy controls, and ROC curves were constructed using the “pROC” package to evaluate the diagnostic efficacy of these key NETs genes. Further validation was carried out using independent datasets to confirm the reliability of the findings.

### TB diagnostic model construction and validation

Using key NETs genes, a nomogram model was constructed through the “rms” package in R. The “total score” reflects the contribution of each predictor variable, with each variable being assigned a corresponding score based on its importance. The model’s accuracy and clinical applicability were rigorously validated through calibration curves, decision curve analysis (DCA), and clinical impact curves.

### Gene set variation analysis

To evaluate the functional differences associated with the upregulation and downregulation of key NETs genes, Gene Set Variation Analysis (GSVA) (Hänzelmann et al., 2013) was performed. GSVA calculates gene set activity scores to identify gene sets associated with distinct biological processes, functions, or pathways. Using KEGG and GO gene sets, the “GSVA” package in R was employed to analyze the enriched pathways associated with the upregulation and downregulation of key NETs genes, revealing their potential biological relevance.

### Immune infiltration analysis

Differences in immune cell infiltration between patients with tuberculosis (TB) and healthy controls were quantified using the CIBERSORT algorithm, a sophisticated deconvolution method that estimates the proportions of 22 immune cell types (e.g., neutrophils, monocytes, T cells) from bulk gene expression data using the LM22 signature matrix. This approach is pivotal for TB research, as it illuminates immune cell dynamics, particularly neutrophil responses associated with neutrophil extracellular traps (NETs).

CIBERSORT analysis was performed on the integrated GEO dataset (GSE54992 and GSE83456, n=150, comprising 80 TB patients and 70 controls) using the CIBERSORT R package (v1.0.0). Data were preprocessed with robust multi-array average (RMA) normalization and ComBat batch correction (sva package, v3.40.0), with genes exhibiting average log2 expression < 0.5 filtered to enhance analytical precision. The analysis employed 100 permutations, and differences in immune cell fractions were evaluated using the Wilcoxon rank-sum test (p < 0.05). Results, visualized through bar and violin plots (ggplot2, v3.3.5), revealed significantly elevated neutrophil and monocyte proportions in TB patients (p < 0.01), consistent with the upregulation of NETs-related genes (AIM2, TNFSF10, C5, IL15, CD274, CYBB).

Validation was conducted using routine blood data from 89 healthy individuals and 150 TB patients, quantifying monocytes, neutrophils, lymphocytes, and eosinophils via automated hematology analyzers (e.g., Sysmex XN-1000). Cell counts, compared using the Mann-Whitney U test (p < 0.05), corroborated elevated neutrophil and monocyte levels in TB patients, aligning seamlessly with CIBERSORT findings.

### Molecular subtype identification of TB based on key NETs genes

Unsupervised hierarchical clustering was conducted on 54 samples from patients with tuberculosis (TB) within the integrated training dataset (GSE54992 and GSE83456) using expression profiles of six pivotal neutrophil extracellular trap (NET)-associated genes (AIM2, TNFSF10, C5, IL15, CD274, CYBB). The ConsensusClusterPlus package (Wilkerson and Hayes, 2010) (version 1.58.0) in R was employed with the following parameters: maxK = 6, reps = 50, pItem = 0.8, pFeature = 1, clusterAlg = “pam” (partitioning around medoids), and distance = “euclidean.” The optimal cluster number (k=2) was determined through the Calinski criterion and inter-cluster correlation analysis, delineating two distinct TB molecular subtypes. Subtype distribution was visualized using t-distributed stochastic neighbor embedding (t-SNE) via the Rtsne package (version 0.16). Differential expression of m6A key regulatory genes across subtypes was evaluated using the Kruskal-Wallis test, with *post-hoc* pairwise comparisons adjusted for multiple testing via the Benjamini-Hochberg method. Results were depicted as heatmaps using the pheatmap package.

### Real-time fluorescence quantitative PCR validation of key NETs genes

Whole blood samples were collected from five TB patients and five healthy volunteers, with informed consent obtained. TB patients were selected based on confirmed diagnosis via clinical features (e.g., sputum smear positivity and/or chest X-ray abnormalities), as detailed in the “Study Population” subsection. Primers were designed using the Primer 3 Plus tool and validated through the NCBI database, with primer sequences listed in **Table 2**. Total RNA was extracted from peripheral blood, followed by cDNA synthesis via reverse transcription. RT-qPCR was conducted according to the kit’s instructions, and relative gene expression was calculated using the 2^-ΔΔCt method to ensure accuracy and reproducibility of the results.

**Table 2:** Primers sequence.

Gene	Primer (5′-3′)	
β-actin	F:TGGCAAAACGTCTTCAGGAGG	R:AGCTTGACTTAGTGGCTTTGG
AIM2	F:TGCGTGCTGATCGTGATCTTC	R:GCTCGTTGGTAAAGTACACGTA
TNFSF10	F:CGGCTCCGACAAGATACTTC	R:TAGGCACGCAGCAAACTC
C5	F:ACAGTCATAGAGTCTACAGGTGG	R:CCAACTGGTCAAGCGAATCTT
IL15	F:TTGGGAACCATAGATTTGTGCAG	R:GGGTGAACATCACTTTCCGTAT
CD274	F:GGACAAGCAGTGACCATCAAG	R:CCCAGAATTACCAAGTGAGTCCT
CYBB	F:TGCCAGTCTGTCGAAATCTGC	R:ACTCGGGCATTCACACACC

### Statistical analysis

In this study, the relationship between continuous variables in two groups was analyzed using the non-parametric Wilcoxon rank-sum test, with P < 0.05 considered statistically significant. All statistical analyses were conducted using R software (version 4.4.1) and Prism 10 (GraphPad Software, USA).

## Results

### Identification of differentially expressed genes

**Figure 2:**
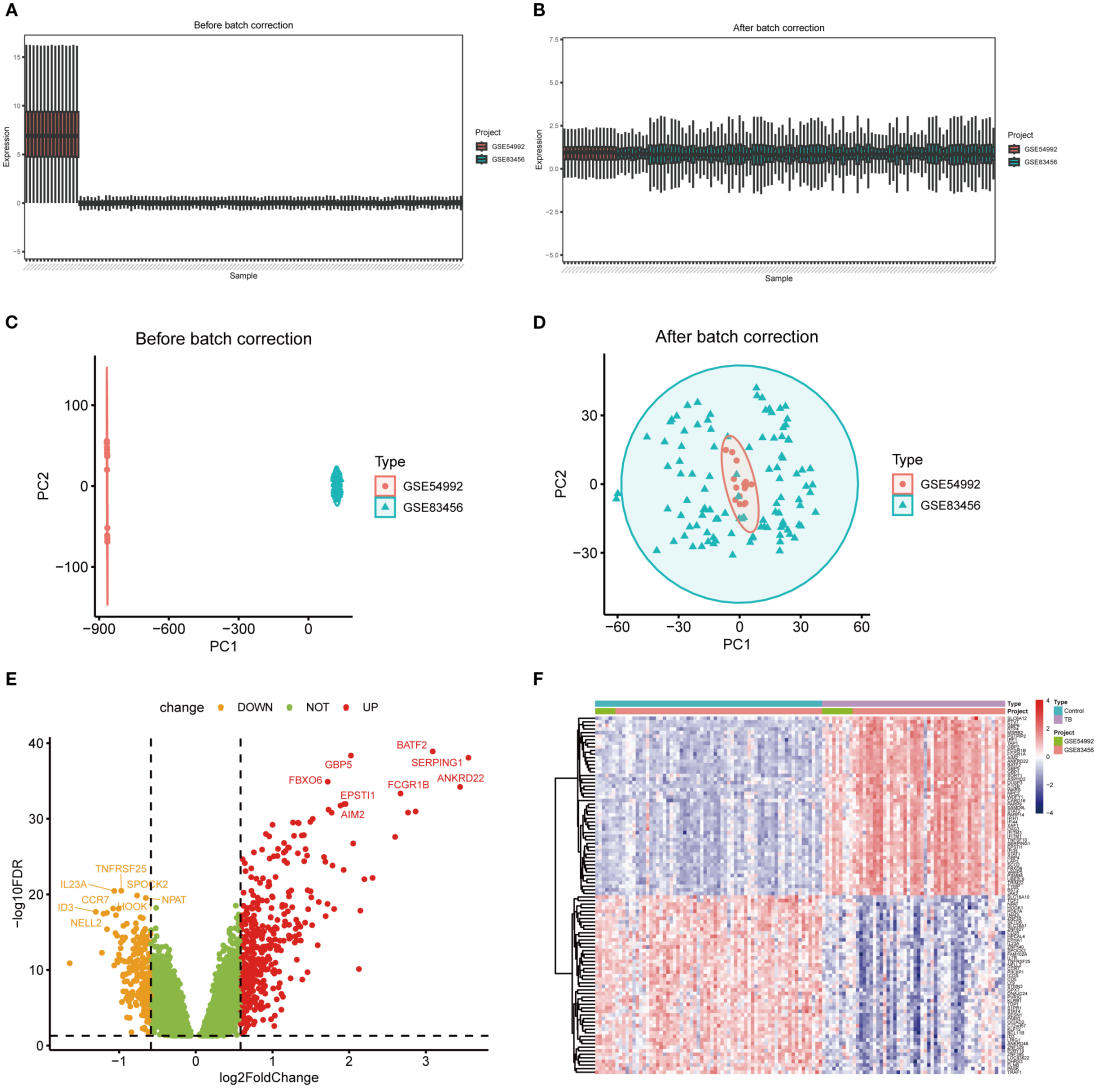
Differentially expressed genes between TB and healthy control samples. **(A, B)** Boxplots illustrating gene expression distributions across two independent datasets before and after batch effect correction, demonstrating reduced batch-associated variability post-correction. **(C, D)** Principal component analysis (PCA) scatterplots showing clear separation of samples by dataset prior to correction and marked integration ost-correction. **(E)** Volcano plot of differentially expressed genes (DEGs), with red and orange dots representing significantly upregulated and downregulated genes, respectively, and green dots indicating non-significant genes. **(F)** Heatmap of DEGs depicting expression patterns across samples and highlighting differences between experimental groups.

To mitigate batch effects between the GSE54992 and GSE83456 datasets, the Combat method was employed for data normalization; the outcomes prior to and following normalization are illustrated in **Figures 2A–D**. Following data integration, a training dataset was assembled comprising 54 TB patient samples and 67 healthy control samples. Applying the selection criteria (|log2 fold change| > 0.585, p < 0.05), a total of 630 DEGs were discerned, including 417 upregulated and 213 downregulated genes (**Figure 2E**). For visual representation, a heatmap depicting the top 50 most significantly altered genes was generated (**Figure 2F**).

### Construction of TB co-expression network and identification of key NETs genes

**Figure 3:**
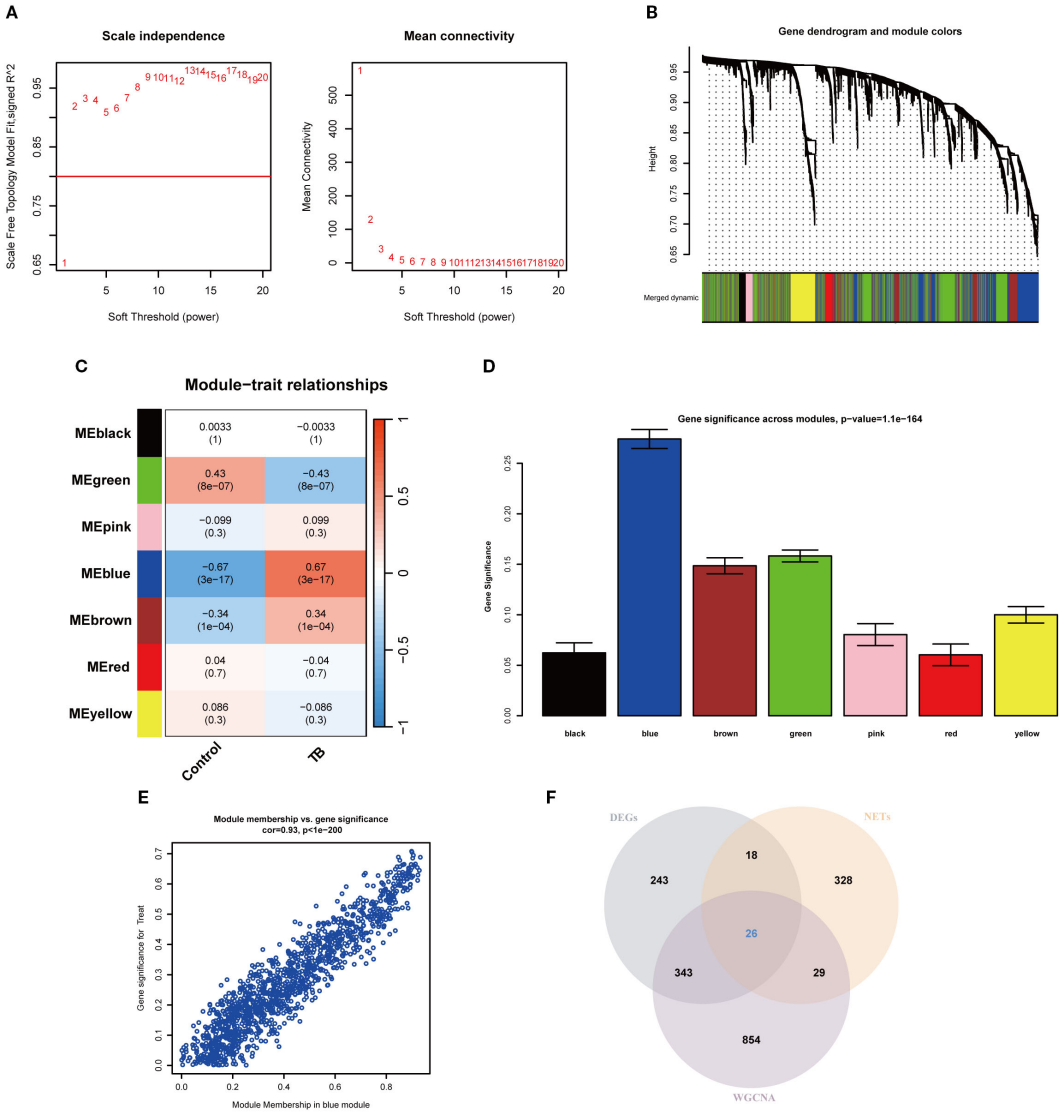
Weighted gene co-expression network analysis and identification of key NETs genes. **(A)** Soft-thresholding power analysis illustrating the selection of the optimal power parameter based on scale-free topology model fit index for network construction. **(B)** Gene dendrogram and module assignment, with distinct colors indicating different co-expression modules identified from hierarchical clustering. **(C)** Heatmap showing the relationship between modules and TB features. **(D)** Bar plot showing gene significance across modules, highlighting the blue module as most significantly associated with the trait. **(E)** Scatter plot depicting the high correlation between module membership and gene significance within the blue module (cor=0.93, p < 1e-200). **(F)** Venn diagram illustrating the overlap among differentially expressed genes (DEGs), tuberculosis-related genes (NETs), and WGCNA-identified module genes.

To precisely pinpoint central genes intimately linked to TB phenotypes, the WGCNA algorithm was employed to construct a comprehensive gene co-expression network. In this analysis, a soft threshold of β = 2 was selected to attain a scale-free topology for the network (refer to **Figure 3A**). Hierarchical clustering was subsequently performed to generate a dendrogram delineating gene modules, which identified seven modules exhibiting analogous gene expression patterns (**Figure 3B**). Ultimately, the blue module emerged as the module most strongly correlated with TB phenotypes, encompassing 1,252 genes, with a correlation coefficient of 0.67 between the module and the clinical phenotype, and a significant p-value of 3 × 10^-6^. The significance of the module genes attained an extraordinary p-value of 1.1 × 10^-^¹^64^ (**Figures 3C–D**). Moreover, a scatter plot corroborated the robust correlation among genes within the blue module, revealing a correlation coefficient of 0.93 and a p-value of 1 × 10^-^²^00^ (**Figure 3E**). Furthermore, by intersecting the DEGs, NRGs, and the pivotal genes derived from the blue module, a total of 26 key NETs genes were identified (**Figure 3F**).

### Functional enrichment analysis of key NETs genes

**Figure 4:**
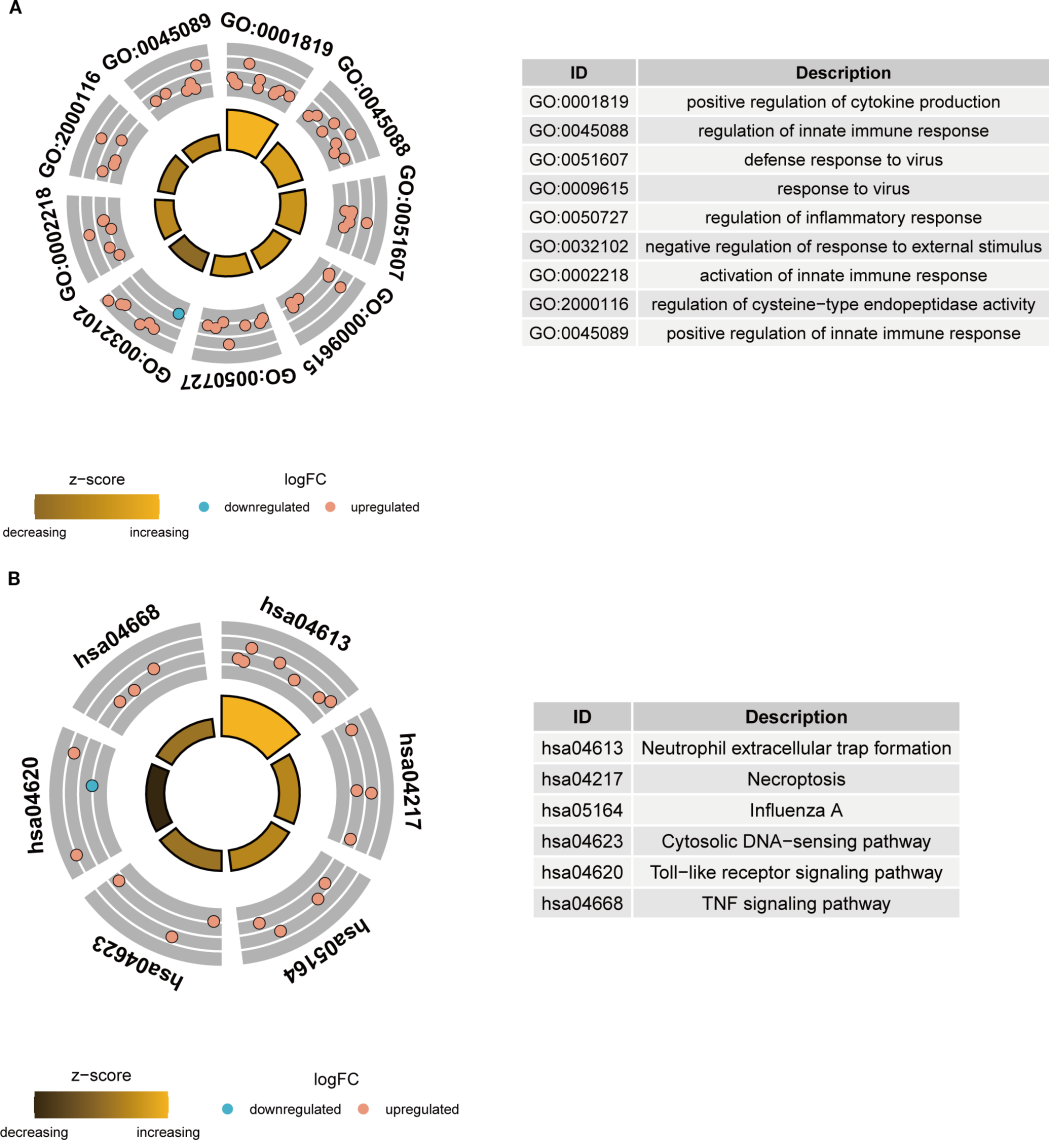
Functional enrichment analysis of key NETs genes. **(A)** GO enrichment analysis circle plot of key NETs genes. **(B)** KEGG enrichment analysis circle plot of key NETs genes.

To elucidate the functional roles and relevance of these key NETs genes in TB, GO and KEGG pathway enrichment analyses were performed on the 26 key genes. GO analysis revealed that these genes predominantly participate in immune-related biological processes, including the positive regulation of cytokine production, modulation of innate immune responses, and orchestration of inflammatory responses (**Figure 4A**). KEGG pathway analysis demonstrated significant enrichment of these genes in critical pathways, notably neutrophil extracellular trap formation, Toll-like receptor signaling, and TNF signaling pathways (**Figure 4B**). Collectively, these findings imply that the key NETs genes are intricately linked to TB-associated immune responses and may exert substantial influence on the disease’s pathological progression.

### Selection of key NETs genes

**Figure 5:**
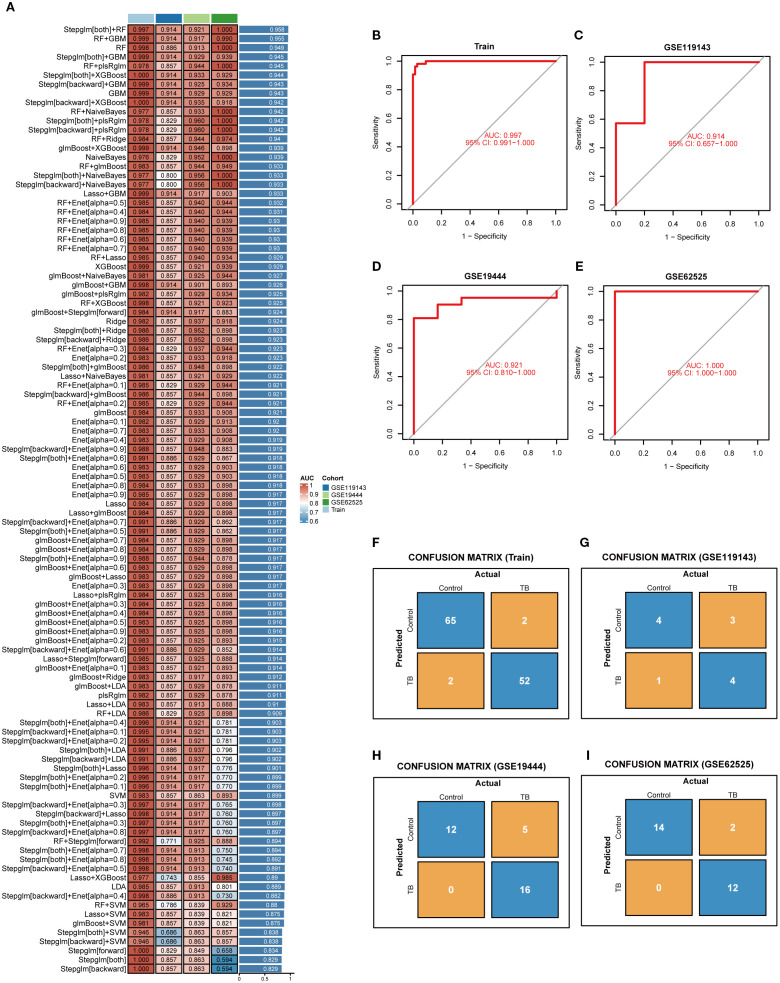
Selection of key NETs genes. **(A)** Evaluation of 113 machine learning algorithm combinations using 10-fold cross-validation. **(B–E)** ROC curves with corresponding AUC values for the selected optimal model in the training set **(B)** and three independent validation datasets (**C–E)**, demonstrating robust sensitivity and specificity. **(F–I)** Confusion matrices for the training set and three validation cohorts, showing classification accuracy and misclassification between control and tuberculosis groups.

In order to identify the most diagnostically potent NETs core genes, 12 machine learning algorithms were employed to develop 113 combined models, utilizing a 10-fold cross-validation framework. Subsequent model evaluation in the training set and three external validation sets indicated that the “Stepglm[both]+RF” algorithm demonstrated the highest predictive performance (**Figure 5A**). Ultimately, employing this algorithm, six key NETs genes were identified, namely AIM2, TNFSF10, C5, IL15, CD274, and CYBB. The AUC values derived from Receiver Operating Characteristic (ROC) analyses for these core genes across four datasets were 0.997, 0.914, 0.921, and 1.00, respectively (**Figures 5B–E**). Furthermore, diagnostic sensitivity and specificity, as determined by confusion matrix analyses, ranged from 96% to 100% and 80% to 100%, respectively, across the datasets (**Figures 5F–I**), underscoring the model’s minimal risk of overfitting and exceptional diagnostic performance.

### Expression and diagnostic value assessment of key NETs genes

**Figure 6:**
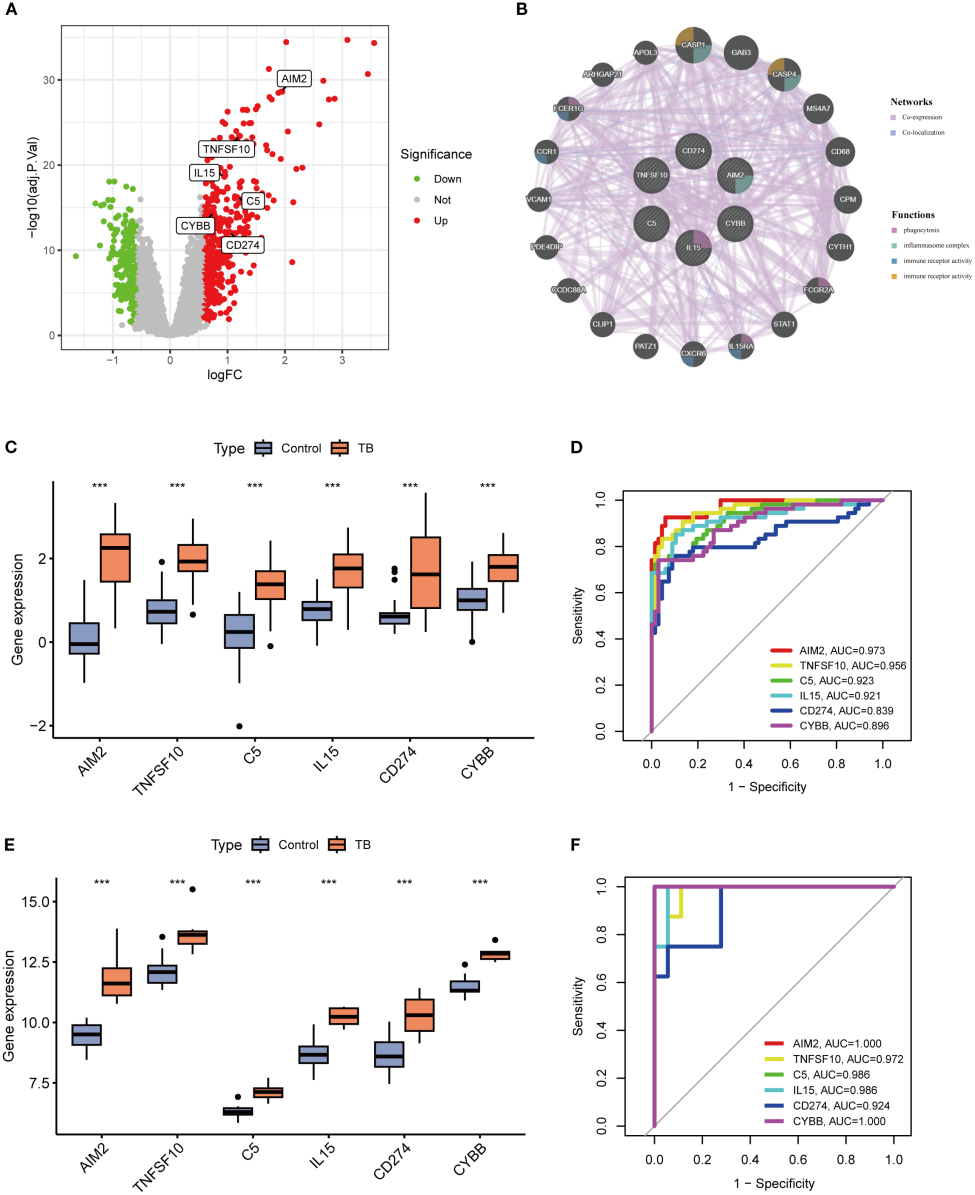
Differential expression analysis of key genes and evaluation of their diagnostic performance. **(A)** Volcano plot illustrating differential expression of key genes between tuberculosis (TB) and control groups. **(B)** Protein-protein interaction (PPI) network of key genes **(C, E)** Boxplots of key gene expression levels in training **(C)** and validation **(E)** cohorts, showing significantly elevated expression in TB versus control groups (***p < 0.001). **(D, F)** Receiver operating characteristic (ROC) curves and area under the curve (AUC) values for key genes in training **(D)** and validation **(F)** datasets, demonstrating high diagnostic accuracy for tuberculosis.

A volcano plot was constructed to visually illustrate the expression profiles of six pivotal neutrophil extracellular trap (NET)-associated genes, revealing significant upregulation of all core genes in patients with tuberculosis (TB) (**Figure 6A**). In bacterial pneumonia, validation of key gene expression showed that only AIM2 and TNFSF10 exhibited statistically significant upregulation, while the other genes did not reach statistical significance (**Supplementary Figure 1**; **Supplementary Material 1**). Subsequent analysis revealed that these genes are predominantly enriched in critical biological processes, such as phagocytosis and inflammasome complex formation (**Figure 6B**). Compared to healthy controls, the expression of these six genes was substantially elevated in TB samples (**Figure 6C**), a finding substantiated in the GSE34608 validation cohort (**Figure 6E**). Receiver operating characteristic (ROC) curve analysis disclosed area under the curve (AUC) values for AIM2, TNFSF10, C5, IL15, CD274, and CYBB of 0.973, 0.956, 0.923, 0.921, 0.839, and 0.896, respectively, with all AUC values in the validation cohort surpassing 0.9, underscoring these genes’ potential as robust diagnostic biomarkers for TB (**Figures 6D, F**).

### Clinical prediction model construction

**Figure 7:**
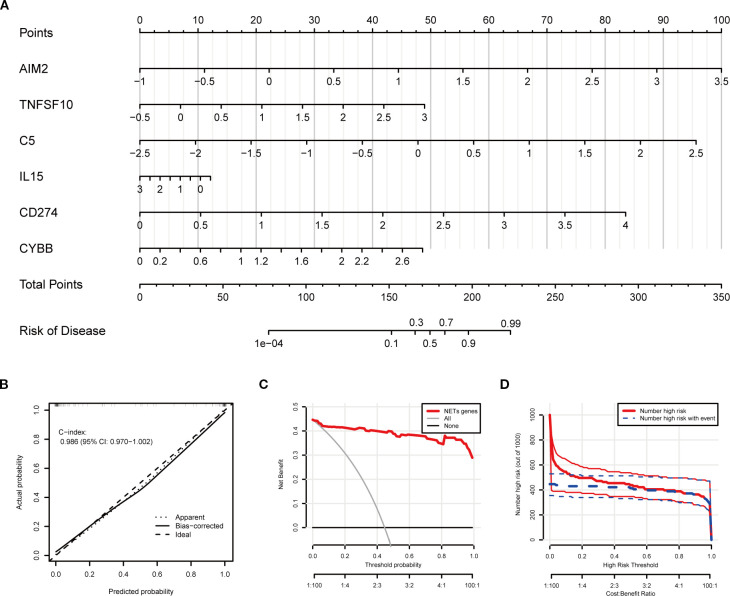
Clinical prediction model construction based on key NETs genes. **(A)** Nomogram integrating expression levels of AIM2, TNFSF10, C5, IL15, CD274, and CYBB for quantitative individualized risk prediction of tuberculosis. **(B)** Calibration curve shows agreement between predicted and observed probabilities, with a concordance index (C-index) of 0.986, indicating excellent predictive accuracy and discrimination. **(C)** Decision curve analysis (DCA) assessing net benefit across different threshold probabilities. **(D)** Plot of the number of patients classified as high risk and number of events at different risk thresholds, demonstrating effective risk stratification by the model.

Using the six key NETs genes, a nomogram model was developed to predict the risk of TB occurrence (**Figure 7A**). The model’s performance was rigorously validated using calibration curves, decision curve analysis (DCA), and clinical impact curves. The calibration curve demonstrated a high degree of consistency between the model’s predictions and actual observations, with a C-index of 0.986 (**Figure 7B**). Decision curve analysis (DCA) indicated that the nomogram model provided a high net benefit, making it appropriate for clinical application (**Figure 7C**). The clinical impact curve further corroborated the strong predictive performance of the model (**Figure 7D**).

### Enrichment analysis of key NETs genes

**Figure 8:**
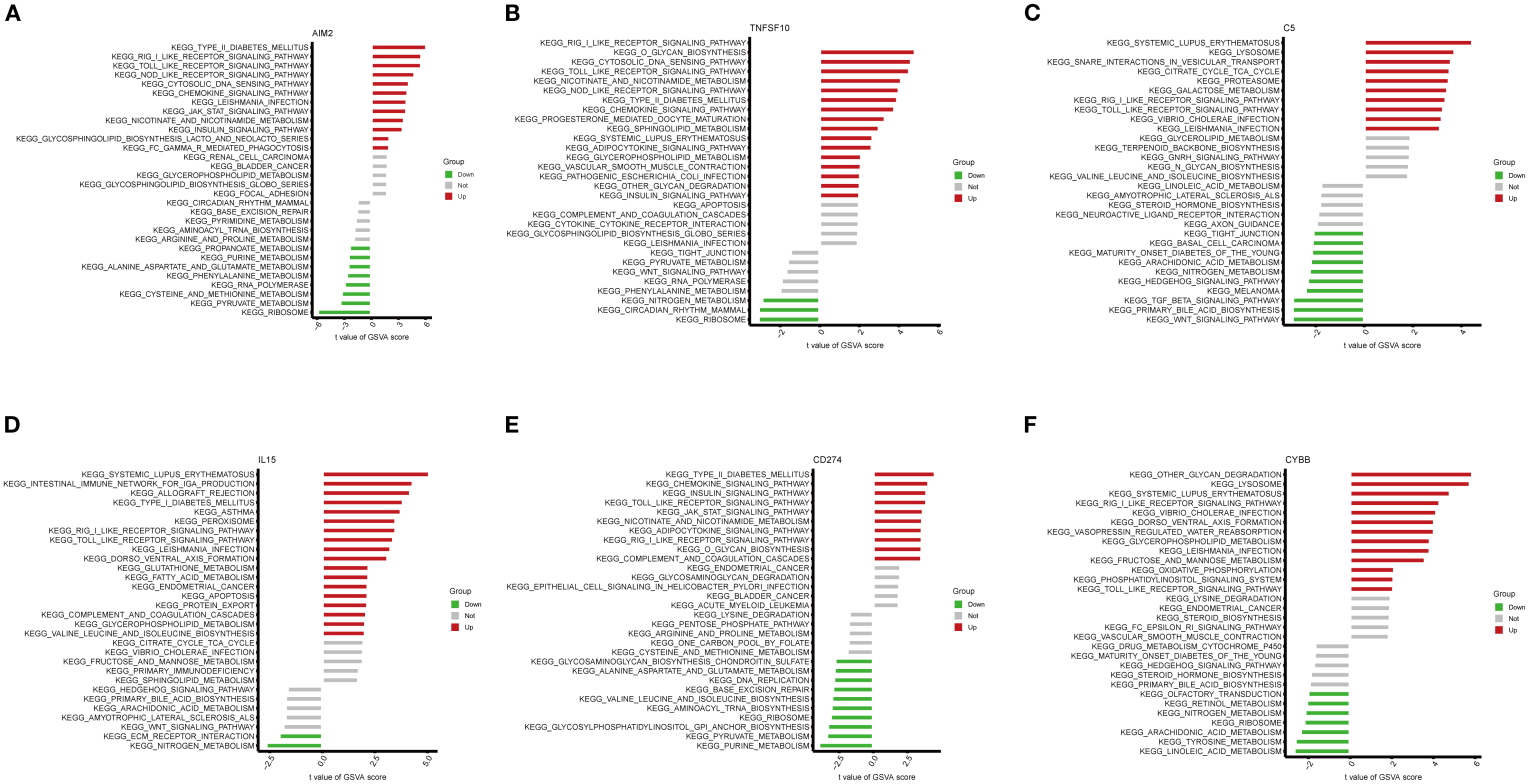
Gene set variation analysis (GSVA) of pathways associated with key genes. **(A–F)** Bar plots depicting KEGG pathway enrichment results related to the key genes AIM2 **(A)**, TNFSF10 **(B)**, C5 **(C)**, IL15 **(D)**, CD274 **(E)**, and CYBB **(F)**.

KEGG pathway enrichment analysis was conducted using GSVA on the six key NETs genes. The findings demonstrated that these genes were primarily involved in key immune-related pathways, including the RIG-I-like receptor signaling pathway, Toll-like receptor signaling pathway, and NOD-like receptor signaling pathway (**Figures 8A–F**). Notably, all six key genes exhibited significant enrichment in the RIG-I-like receptor and Toll-like receptor signaling pathways, suggesting that these genes may impact TB onset and progression by modulating critical immune pathways.

### Immune infiltration correlation analysis

**Figure 9:**
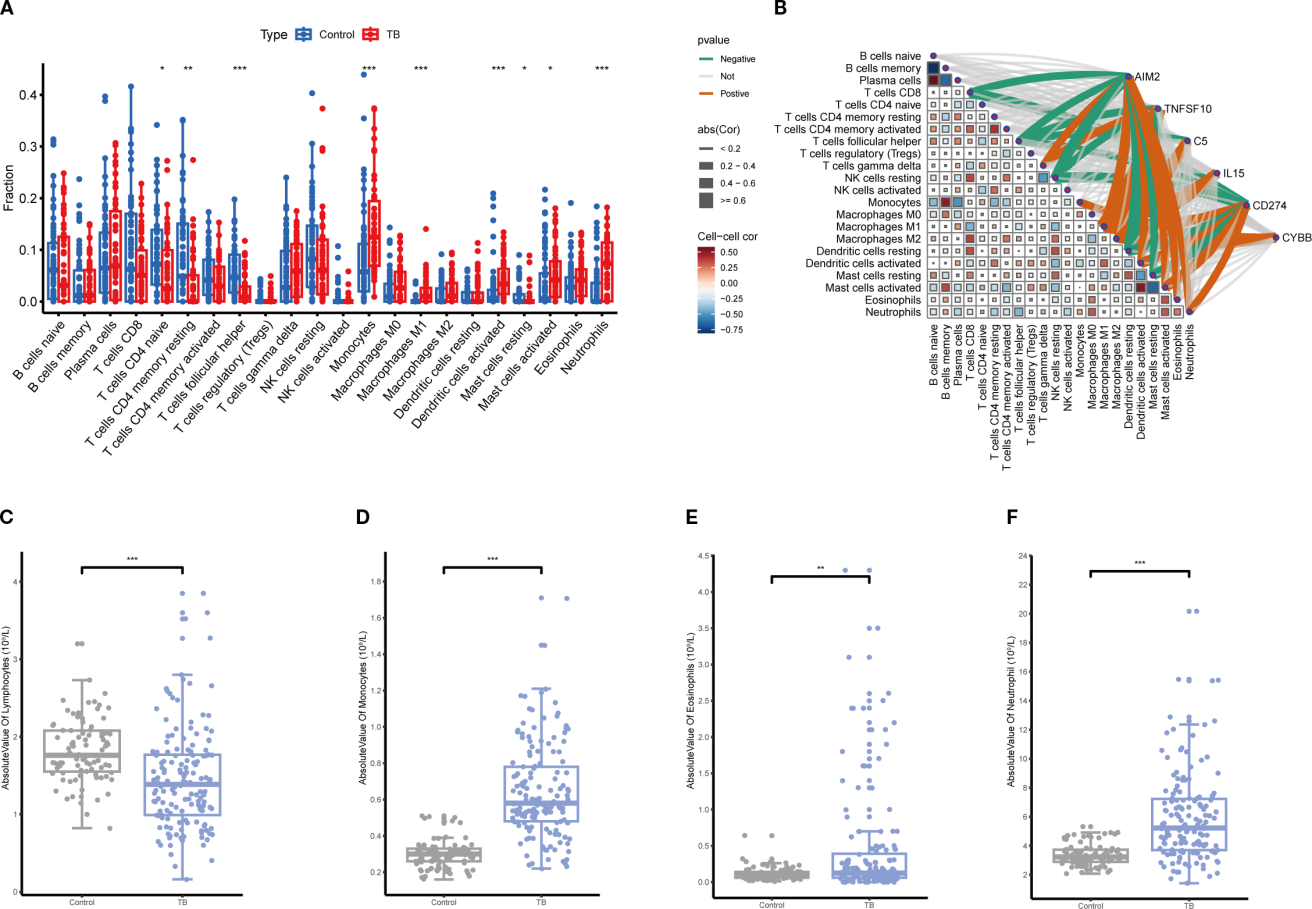
Immune infiltration correlation analysis. **(A)** Comparison of relative abundances of 22 immune cell subtypes between tuberculosis (TB) and control groups based on immune infiltration analysis, with significant alterations observed in multiple immune cell fractions in the TB group (*p<0.05, **p<0.01, ***p<0.001). **(B)** Correlation network between key gene expression and immune cell infiltration levels. **(C–F)** Comparison of absolute counts of lymphocytes **(C)**, monocytes **(D)**, eosinophils **(E)**, and neutrophils **(F)** in peripheral blood showing significant increases in TB patients (**p<0.01, ***p<0.001).

CIBERSORT analysis of immune infiltration characteristics in TB patients and healthy controls revealed a significant increase in monocytes, macrophages, eosinophils, and neutrophils in TB patients compared to healthy controls, while CD4+ and CD8+ T cells were significantly reduced (**Figure 9A**). Further analysis demonstrated that the expression levels of key NETs genes were significantly correlated with the infiltration of various immune cells (**Figure 9B**), indicating that NETs core genes may contribute to TB pathogenesis by modulating the immune microenvironment. The validation analysis of blood routine data similarly showed a significant increase in monocytes and neutrophils in TB patients, with a concomitant decrease in lymphocytes (**Figures 9C–F**), consistent with the immune infiltration analysis findings.

### Identification of NETs-related subtypes in TB

**Figure 10:**
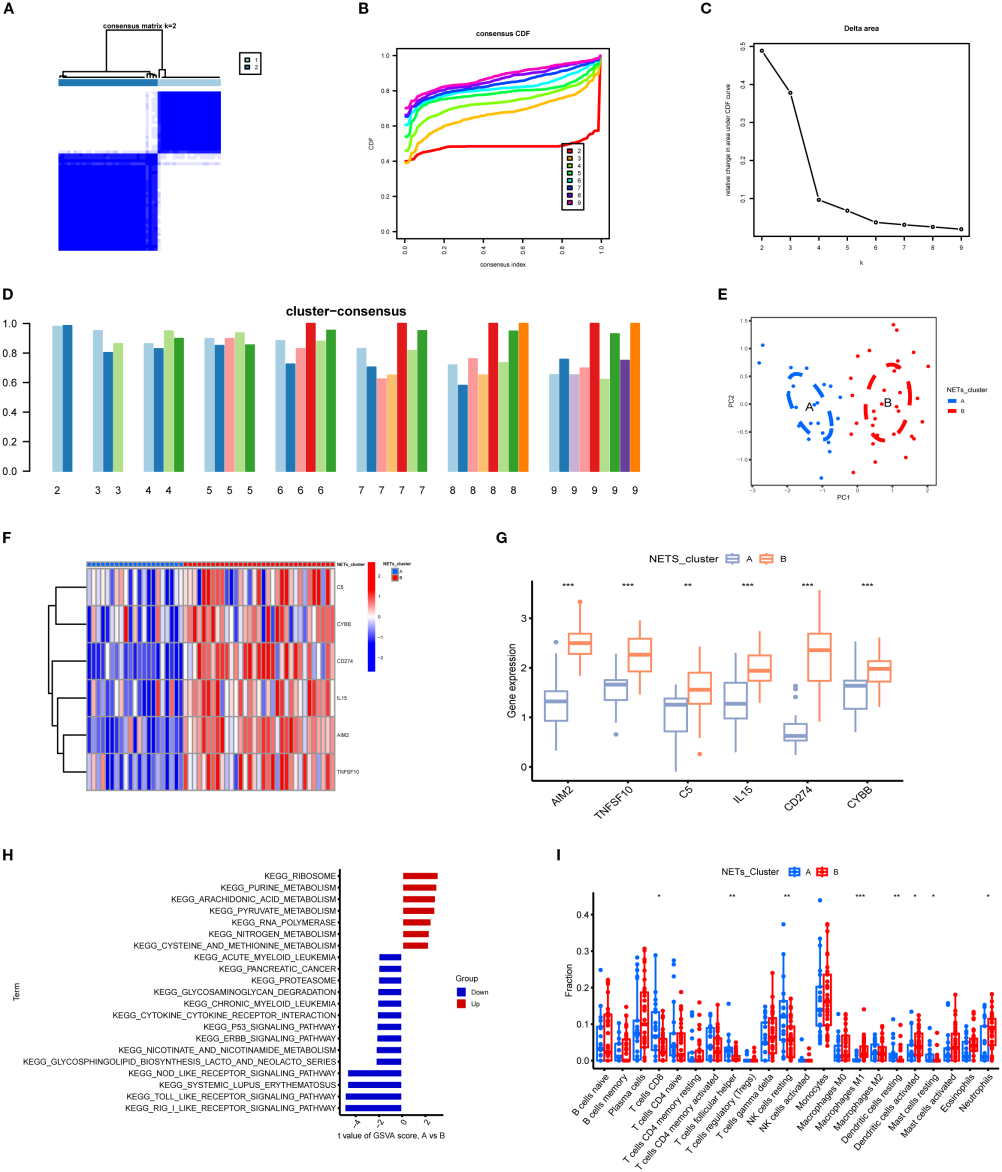
Subgroup classification of tuberculosis samples based on key gene expression patterns and associated functional characterization. **(A)** Consensus clustering matrix at k=2 showing optimal sample clustering stability. **(B)** Cumulative distribution function (CDF) curves for various k values, aiding determination of optimal cluster number. **(C)** Relative change in area under CDF curve indicates marked decrease at k=2, suggesting two distinct clusters. **(D)** Bar plot of cluster consensus scores at different k values. **(E)** Principal component analysis (PCA) scatter plot showing clear separation between two subgroups. **(F)** Heatmap depicting expression profiles of key genes across two clusters, with red and blue representing high and low expression levels, respectively. **(G)** Boxplots showing significant differential expression of key genes between clusters (**p<0.01, ***p<0.001). **(H)** KEGG pathway enrichment based on gene set variation analysis (GSVA) between clusters, highlighting upregulated (red) and downregulated (blue) pathways. **(I)** Immune cell infiltration differences across subgroups, illustrating variations in abundance of diverse immune cell subsets. *P<0.05.

Using the expression data of the six key NETs genes, consensus clustering algorithms were applied to classify 54 TB samples. The optimal number of clusters was determined to be k = 2, with the CDF curve exhibiting minimal fluctuation within the range of a consistency index between 0.4 and 0.6 (**Figures 10A, B**). Between k = 2 and k = 9, the area under the CDF curve demonstrated differences between two consecutive CDF curves (k and k-1) (**Figure 10C**). When k = 2, the consistency score for the subtypes reached its highest value (**Figure 10D**). Principal component analysis (PCA) results revealed that the 54 TB patients could be distinctly classified into Cluster A (n = 21) and Cluster B (n = 33) (**Figure 10E**). Expression differences of the six key NETs genes between Cluster A and Cluster B were evaluated to investigate the molecular characteristics of the clusters. Distinct expression profiles of the NETs core genes were observed between Cluster A and Cluster B (**Figure 10F**). In Cluster B, AIM2, TNFSF10, C5, IL15, CD274, and CYBB exhibited significant upregulation (**Figure 10G**).

To explore the pathway activity and associated biological functions within each cluster, GSVA was applied. The results demonstrated that ribosome function, purine metabolism, and arachidonic acid metabolism were significantly enriched in Cluster A, whereas Cluster B showed notable enrichment in RIG-I-like receptor signaling pathways, Toll-like receptor signaling pathways, and NOD-like receptor signaling pathways (**Figures 10H**). These enrichment results imply that Cluster B is more strongly associated with the NETs core genes and the pathological processes of TB. Moreover, immune cell infiltration analysis revealed significant differences in the immune microenvironment between Cluster A and Cluster B (**Figure 10I**). The abundance of M0 macrophages, eosinophils, and neutrophils was significantly higher in Cluster B, whereas CD8+ T cell abundance was significantly lower, indicating that TB patients in Cluster B are more prone to increased immune cell infiltration.

### Validation of NETs core gene expression in clinical samples

**Figure 11:**
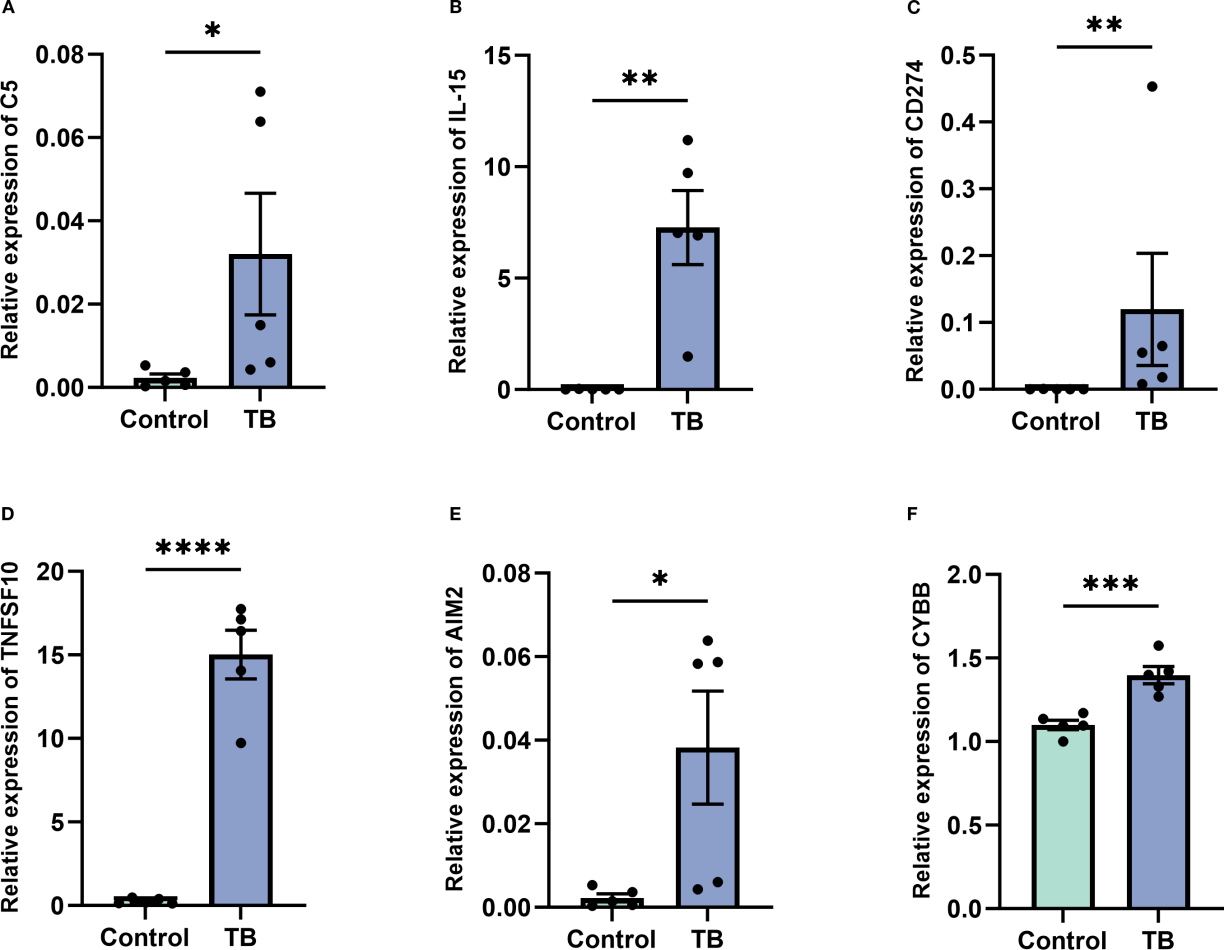
Validation of key gene expression in tuberculosis (TB) patients versus controls. **(A–F)** Quantitative real-time PCR analysis demonstrating significantly elevated expression levels of key genes C5 **(A)**, IL-15 **(B)**, CD274 **(C)**, TNFSF10 **(D)**, AIM2 **(E)**, and CYBB **(F)** in peripheral blood samples from TB patients compared to healthy controls. Data are presented as mean ± standard error of the mean (SEM). Statistical significance is denoted as *p < 0.05, **p < 0.01, ***p < 0.001, and ****p < 0.0001.

To further validate the expression of the six NETs core genes, RT-qPCR was conducted on clinical samples from five TB patients and five healthy controls. The results demonstrated that these genes were significantly upregulated in TB patients, consistent with the bioinformatics analysis findings (**Figures 11A–F**).

## Discussion

The pathogenesis of tuberculosis (TB) is marked by a distinctive feature: Mycobacterium tuberculosis (MTB), the etiological agent, can survive for prolonged periods in diverse intracellular environments, thereby maintaining a persistent infection. Early infection with this pathogen frequently does not manifest with overt clinical symptoms, instead revealing subtle alterations at the molecular genetic level. Despite its latent nature, this infection continues to pose a substantial threat to host health, especially when the immune system encounters external stress or regulatory imbalances, which may prompt the infection to rapidly progress into active disease (Boom et al., 2021; Chandra et al., 2022). Existing literature suggests that the function of neutrophil extracellular traps (NETs) is intricately linked to the onset and progression of TB (Cavalcante-Silva et al., 2023). However, the precise molecular genetic mechanisms by which NETs influence the pathogenesis and progression of TB remain to be fully elucidated. Therefore, this study seeks to investigate the role of NETs-related regulatory genes (NRGs) in TB, offering new theoretical insights for molecular pathological research on TB and laying the foundation for future diagnostic and therapeutic strategies.

In this study, we integrated the GSE54992 and GSE83456 datasets from the GEO database to perform a comprehensive analysis of gene expression levels in healthy controls and TB patients. A total of 630 differentially expressed genes (DEGs) were identified, of which 417 were significantly upregulated and 213 significantly downregulated. To further refine our identification of key genes associated with TB clinical phenotypes, we utilized Weighted Gene Co-expression Network Analysis (WGCNA) to construct a co-expression network for TB samples. The results revealed that the blue module, among the seven identified gene modules, exhibited a significant correlation with TB. This module comprised 1,252 genes. In subsequent analyses, by intersecting the DEGs, blue module genes, and NETs-related genes, we identified 26 pivotal NRGs. To gain a deeper understanding of these genes’ functions, we annotated the NRGs using functional enrichment analyses (GO and KEGG), uncovering their potential biological roles in TB.

GO analysis revealed that these key genes were predominantly involved in processes related to innate immune regulation, defense responses, and viral infections, implying that these genes may play a critical role in the host immune response to Mycobacterium tuberculosis. Concurrently, KEGG analysis further demonstrated that these key genes were significantly enriched in crucial immune pathways, such as neutrophil extracellular trap formation, Toll-like receptor signaling pathways, and TNF signaling pathways. Previous studies have shown that chronic viral infections can exacerbate Mycobacterium tuberculosis co-infections (Xu et al., 2021), suggesting that the host’s immune response to TB may resemble responses to viral infections. Yan et al. further substantiated this idea, positing that the human immune response to MTB aligns more closely with a viral infection pattern than with a typical bacterial infection (Wang et al., 2024). Furthermore, Toll-like receptors, as essential components of innate immunity, play a pivotal role in the host’s defense against MTB (Saraav et al., 2014). The findings of this study not only reinforce these existing conclusions but also further elucidate that the identified NRGs play a central role in the immune response following Mycobacterium tuberculosis infection, underpinned by the high accuracy of the gene selection method employed.

In the subsequent screening process, we conducted a comprehensive analysis of the 26 NETs-related genes (NRGs) using 113 machine learning algorithms and ultimately identified six core NRGs through the “StepgIm[both]+RF” combination algorithm, including AIM2, TNFSF10, C5, IL15, CD274, and CYBB. These core genes were further validated in the GSE34608 dataset, exhibiting expression levels consistent with those observed in the training set. These six genes were significantly upregulated in TB patients and exhibited excellent diagnostic performance in the validation set, with ROC curve AUCs exceeding 0.90, underscoring their substantial potential in TB diagnosis.While our model demonstrated exceptionally high AUC values across multiple retrospective cohorts, we acknowledge that such performance is uncommon and must be interpreted with caution. The definitive validation of its diagnostic and prognostic utility requires evaluation in a large-scale, independent, and prospectively collected clinical cohort before it can be considered for clinical translation.

AIM2, a cytoplasmic sensor protein, plays a pivotal role in inflammatory responses by detecting double-stranded DNA (dsDNA) from damaged cells, inducing cytokine expression that drives the development of inflammatory diseases (Hornung et al., 2009). MTB infection activates the AIM2 inflammasome, increasing the pathogen burden and exacerbating the infection (Qu et al., 2020). Tumor necrosis factor-related apoptosis-inducing ligand (TNFSF10) induces apoptosis through death receptors TRAILR1/DR4 and TRAILR2/DR5 (Cardoso Alves et al., 2021). Predominantly expressed on immune cells, TNFSF10 is utilized by cytotoxic T cells and NK cells to eliminate target cells. MTB cell wall components in neutrophils release soluble TNFSF10, which regulates intracellular pathogen infection (Kuribayashi et al., 2008). Type 2 cytokines in TB patients also aid anti-infection processes via TNFSF10-dependent mechanisms (Caccamo et al., 2015). Elevated serum TNFSF10 levels in TB patients further support these findings (La Manna et al., 2018).

Complement component C5 is crucial for immune defense. MTB-infected macrophages secrete C5, which cleaves into C5a peptides, modulating IL-12 secretion and activating T cells to produce IFN-γ, enhancing macrophage bactericidal activity (Carter and Murphy, 1999). While MTB proliferates in unactivated macrophages, IFN-γ-activated macrophages generate nitric oxide to eradicate pathogens (Adams et al., 1993). In MTB-infected mouse models, C5-deficient mice are more susceptible to severe TB infections (Mashruwala et al., 2011).

IL15, produced primarily by myeloid cells, bridges innate and adaptive immunity. It regulates T cell activity, activates NK cells, and enhances dendritic cells and macrophages (Patidar et al., 2016). Upregulated IL15 in dendritic cells induces IFN-γ, inhibiting MTB growth in macrophages via the IFN-γ-NO axis (Kwon et al., 2019). CD274 (PD-L1), an immune checkpoint molecule, may be upregulated in MTB infection, contributing to immune evasion by expanding regulatory T cells (Shi et al., 2022). CD274 is also a potential target for TB diagnosis and treatment (Long et al., 2021; Yang et al., 2023).

CYBB encodes gp91-phox, a component of the phagocyte oxidase complex that generates superoxide and reactive oxygen species (ROS) with bactericidal effects (Frazão et al., 2015). However, excessive ROS production can damage host tissues, exacerbating TB symptoms (Ma et al., 2023). Upregulation of CYBB may reflect its involvement in inflammation-related tissue damage. Experimental validation confirmed that the expression of these six genes was significantly higher in TB patients compared to healthy controls, consistent with GEO dataset results.

A nomogram model based on the six NETs core genes was constructed to demonstrate their diagnostic capability. Calibration curves confirmed the model’s robust predictive ability in validation datasets, underscoring the clinical potential of these genes. Thus, AIM2, TNFSF10, C5, IL15, CD274, and CYBB emerge as promising targets for TB diagnosis and treatment. Their pivotal roles in host immune responses offer novel insights into TB pathogenesis and inform the development of precise diagnostic tools and targeted therapies.

GSVA enrichment analysis revealed that the six NETs core genes are significantly enriched in the RIG-I-like and Toll-like receptor signaling pathways. These pathways are initiated by pattern recognition receptors that detect exogenous nucleic acids (Zhou et al., 2017) and subsequently activate cascades inducing pro-inflammatory cytokines, chemokines, and type I interferons that mediate adaptive immune responses. Moreover, these receptors regulate autophagy, bridging innate and adaptive immunity (Deretic, 2012). Thus, NETs core genes may significantly influence TB pathogenesis and progression by modulating these pathways, offering new insights into TB pathology.

TB progression is intricately linked to the host immune response elicited by Mycobacterium tuberculosis (Mtb) infection (Liu et al., 2017). Effective TB treatment requires precise immune regulation. Using the CIBERSORT algorithm, we evaluated immune cell infiltration in TB patients versus healthy controls and correlated these patterns with NETs core gene expression. TB patients exhibited reduced CD4^+^ T cells and elevated monocytes, neutrophils, and eosinophils compared to healthy controls. These findings were corroborated by blood routine data from 89 controls and 150 TB patients. Overall, the immune alterations—elevated monocytes, neutrophils, and eosinophils with reduced lymphocytes—underscore their association with TB pathogenesis.

Monocytes, key innate immune cells, differentiate into macrophages upon tissue migration. MTB infects macrophages and proliferates within them, driving TB progression (Sun et al., 2022). Neutrophils, potential markers of TB severity, have garnered increasing attention and are emerging as targets for host-directed therapies in TB (Nwongbouwoh Muefong et al., 2022). Effective T cell-mediated adaptive responses are essential for controlling MTB infection, and their dysfunction may exacerbate TB (Wufuer et al., 2023). Analysis of immune cell infiltration and NETs core gene expression revealed significant correlations with immune-related pathways, suggesting that these genes modulate immune responses in TB.

This study elucidates the potential immune regulatory roles of NETs core genes via the RIG-I-like receptor and Toll-like receptor signaling pathways, as well as their significant association with immune cell infiltration. This not only furnishes a novel perspective on the pathological mechanisms of tuberculosis (TB) but also underpins the development of immune regulation-based diagnostic and targeted therapeutic strategies.

Utilizing the expression profiles of six NETs core genes, this study employed non-negative matrix factorization (NMF) clustering analysis to stratify TB patients into two distinct groups, designated A and B. In Group B, these core genes exhibited markedly higher expression levels that were positively correlated with the extent of neutrophil infiltration. Moreover, in Group B, these genes were significantly enriched in immune-related pathways—including the RIG-I-like and Toll-like receptor signaling pathways—a distinction absent in Group A. This observation implies that Group B patients may exhibit a more pronounced inflammatory response and a heightened propensity for NETs formation, potentially portending a more adverse clinical prognosis. Given tuberculosis’ well-documented clinical heterogeneity and variable therapeutic responses, these findings underscore the necessity for precise patient stratification and optimized treatment management. Hence, the results not only yield critical biological insights into the diverse clinical phenotypes of TB but also establish a foundation for future personalized and targeted intervention strategies.

Notwithstanding its significant findings, this study is subject to several limitations. First, the small sample size in the RT-qPCR validation restricts the statistical power; future studies with larger, more diverse cohorts are warranted to confirm generalizability. Second, our NETs-related gene set, derived from current literature and databases, is inherently a snapshot of a rapidly evolving field. Consequently, it is subject to the evolving understanding of NETs biology and potential selection bias inherent in knowledge-based compilations. Future work should involve refining this set as new mechanistic discoveries emerge and integrating data-driven approaches to identify novel candidate genes. Third, a significant limitation is the reliance on public GEO datasets, which predominantly feature populations from specific geographic and ethnic backgrounds. Therefore, the universal applicability of our proposed biomarkers, particularly in high-burden regions like sub-Saharan Africa and South Asia, has not been established and requires dedicated validation in these populations. Fourth, as this study is predicated upon bioinformatics and expression data, it lacks direct functional validation through cellular or animal models. Consequently, the mechanistic insights presented remain preliminary, and further investigations incorporating *in vitro* and *in vivo* experiments are warranted to definitively establish the causal roles of the identified genes in TB pathogenesis.

## Conclusion

Building on these findings, this study represents the first comprehensive exploration of the molecular attributes of neutrophil extracellular trap (NET)-associated genes (NRGs) in patients with tuberculosis (TB), identifying six prospective biomarkers—AIM2, TNFSF10, C5, IL15, CD274, and CYBB. Clustering analysis based on these pivotal genes effectively delineated TB patients into two subgroups with distinct molecular signatures. These results indicate that these NET-associated core genes exert a critical regulatory influence on the initiation and progression of TB, presenting promising biological targets for early diagnosis and establishing a robust theoretical framework for personalized therapeutic strategies.

To further elucidate the clinical applicability of these six genes, they can be incorporated into a clinical real-time fluorescence quantitative PCR (RT-qPCR) assay, involving RNA extraction from peripheral blood, cDNA synthesis, and amplification using gene-specific primers (**Table 2**). Expression levels, quantified via the 2^-ΔΔCt method and normalized to a housekeeping gene (e.g., GAPDH), can be integrated into a composite gene expression score through logistic regression or machine learning models. A diagnostic threshold, established via receiver operating characteristic (ROC) curve analysis, can distinguish TB patients from healthy controls with optimized sensitivity and specificity. This methodology aligns with established TB biomarker research (Anderson et al., 2014). Future investigations should validate these biomarkers in larger, more diverse cohorts to refine diagnostic thresholds and explore their integration into point-of-care diagnostic platforms, thereby advancing precision medicine in TB management.

## Data Availability

The original contributions presented in the study are included in the article/supplementary material. Further inquiries can be directed to the corresponding author.
