# Supplementary material for: Machine learning-driven discovery of NETs-associated diagnostic biomarkers and molecular subtypes in tuberculosis
Source: Front Cell Infect Microbiol. 2025 Oct 1;15:1591464. doi: 10.3389/fcimb.2025.1591464 (PMC12521227; doi:10.3389/fcimb.2025.1591464)
Supplement: Supplementary file 1 [file DataSheet1.pdf]

泗阳医院科研伦理委员会伦理审查批件

伦理审查编号：HY2024005

项目全称	基于 RNA-seq 和机器学习分析结核病的诊断生物标志物及免疫浸润		
项目负责人	蔡子涵	科室	检验科
职称	初级检验师	项目编号	2024SY005
联系方式	15751519947	审查方式	☑会议审查　□快速审查
审查日期	2024年7月18日	审查地点	第四会议室
审查结果	委员应到人数　9 人	委员实到人数	9 人
	同意9 票	不同意0票	做必要的修正后同意0票
	做必要的修正后重审 0票	终止或暂停试验 0票	
审查意见	根据国家卫计委《涉及人的生物医学研究伦理审查办法》（2023）、国家食品药品监督管理总局《药物临床试验质量管理规范》（2020）、《药物临床试验伦理审查工作指导原则》（2010）、WMA《赫尔辛基宣言》和 CIOMS《人体生物医学研究国际道德指南》等的伦理原则，经本伦理委员会审查：<br>**同意** 按临床研究方案进行 基于 RNA-seq 和机器学习分析结核病的诊断生物标志物及免疫浸润 研究<br>该项目进行中如发生下列情况，须书面报告本伦理委员会：<br>1）对临床方案、知情同意书等的任何修改；<br>2）更换主要研究者；<br>3）发生严重不良事件；<br>4）出现任何可能显著影响试验进行或增加受试者危险的情况；<br>5）出现违反方案情况；<br>6）暂停或提前终止临床研究。<br>该项目完成后，请向本伦理委员会提交结题报告。		
批件有效期	批件有效期为三年，超过效期请继续提出申请；如该项目在批件有限期内未能启动临床研究，本批件作废，需要重新提交伦理审查申请。<br>本伦理委员会将会对该项目跟踪审查（**跟踪审查频率：12 个月**）。		
联系人	刘芳	联系电话	0527-80626502
主任委员签字	[illegible]	批准日期	2024-7-18
委员签名	[illegible]		

泗阳医院伦理委员会（盖章）

日期：2024年7月18日

Ethics examination and approval document of Scientific Research

Ethics Committee of Siyang Hospital

Ethical review number：HY2024005

**Full name of the project**	Analysis of diagnostic biomarkers and immune infiltration of tuberculosis based on RNA-seg and machine learning			
**Project leader**	Zihan Cai	**Section**	Clinical laboratory	
**The title of a professional post**	Junior surveyor	**Item number**	2024SY005	
**Contact information**	15751519947	**Review mode**	Conference review；Expedited review	
**Review date**	July 18, 2024	**Place of review**	Conference Room 4	
**Review result**	The number of members should be present	9	The number of members is confirmed	9
	Yes by 9 votes	Dissenting 0 votes	Agreed by 0 votes with the necessary amendments	
	Retrial 0 votes after necessary amendment	Termination or suspension of the trial 0 votes		
**Review opinion**	In accordance with the National Health and Family Planning Commission "Measures for Ethical Review of Biomedical Research involving Humans" (2023), the State Food and Drug Administration "Quality Management Standards for Drug Clinical Trials" (2020), "Guidelines for Ethical Review of Drug Clinical Trials" (2010), WMA Declaration of Helsinki and CIOMS "International Ethics for Human Biomedical Research. Guidelines and other ethical principles, reviewed by this Ethics Committee:<br>Agreed to conduct a diagnostic biomarker and immunoinfiltration study of tuberculosis based on RNA-seg and machine learning analysis according to the clinical study protocol<br>In the course of the project, any of the following situations shall be reported to the Ethics Committee in writing:<br>1) Any modification of clinical protocol, informed consent, etc.;<br>2) Change the principal investigator;<br>3) Serious adverse events occur;<br>4) Any situation that may significantly affect the conduct of the test or increase the risk to the subject:<br>5) Violation of the scheme;<br>6) Suspension or early termination of clinical studies.<br>After the completion of the project, please submit the final report to the Ethics Committee.			
**Validity period of approval**	The approval document is valid for three years, please continue to apply after the validity period; If the project fails to initiate clinical study within the limited period of approval, this approval is invalid and a new application for ethical review is required. The Ethics Committee will conduct follow-up review of the project (**follow-up review frequency :12 months**).			
**Contact person**	Fang Liu	**Contact number**	0527-80626502	
**Signature of the chairman**	Xiuting Wang	**Date of approval**	July 18, 2024	
**Member's signature**				

Siyang Hospital Ethics Committee (Seal)

Date: July 18, 2024
