# Supplementary material for: Machine learning-driven discovery of NETs-associated diagnostic biomarkers and molecular subtypes in tuberculosis
Source: Front Cell Infect Microbiol. 2025 Oct 1;15:1591464. doi: 10.3389/fcimb.2025.1591464 (PMC12521227; doi:10.3389/fcimb.2025.1591464)
Supplement: Supplementary file 2 [file DataSheet2.pdf]

id	logFC	AveExpr	t	P.Value	adj.P.Val	B
RASGRP1	-1.466741	8.1031731	-13.44164	1.2076908	4.5626561	20.6226731774615
CD96	-1.214979	8.0183002	-13.04201	2.4923135	4.7079802	19.9726900793442
IL2RB	-2.212342	9.3108147	-12.54718	6.2533086	7.8749999	19.1415292203156
HS.276860	-1.06775	8.7904636	-12.31296	9.7533292	8.9333648	18.7376559994521
BCL11B	-1.691603	9.1834261	-12.21253	1.1822875	8.9333648	18.5623750367785
UQCRQ	2.0387521	9.1814959	12.051021	1.6148195	1.0167980	18.2778311710262
PYHIN1	-1.571979	8.2880451	-11.67378	3.3833042	1.7136397	17.6001369824571
MRPL23	1.0587850	8.6028603	11.638488	3.6286706	1.7136397	17.5357973926526
BRI3	1.1638765	11.045241	11.126500	1.0185893	3.9153320	16.5836760983148
NUP214	1.5731517	10.736240	11.053212	1.1837626	3.9153320	16.4444972302377
RNF216	-0.956148	8.2761397	-11.04315	1.2084956	3.9153320	16.425335805349
TGFBR3	-1.883044	9.0893808	-11.02922	1.2436205	3.9153320	16.3987825585719
CD247	-1.746102	11.437898	-10.95458	1.4505985	4.2156626	16.2560194691453
LSMD1	1.0005034	7.3652313	10.918393	1.5633586	4.2188350	16.1865481674265
SKAP1	-1.757967	9.4701545	-10.81766	1.9272370	4.8540676	15.9921868526723
FBXO21	-0.830472	8.2035975	-10.78459	2.0648451	4.8756156	15.9280709709027
ETS1	-1.455515	11.091444	-10.67579	2.5932259	5.7630632	15.7160597731761
GIMAP1	-1.228478	8.7848408	-10.58067	3.1685465	6.6504271	15.5293739638986
NDUFB7	1.3026038	8.5826436	10.245067	6.4835986	1.2892124	14.8604774195252
PRKCH	-1.418161	9.6280764	-10.14216	8.0984199	1.5192718	14.6521624032366
ITK	-1.333637	9.4472443	-10.12284	8.4448674	1.5192718	14.6128957489016
SPOCK2	-1.902926	10.956437	-10.08724	9.1238809	1.5668191	14.540378771002
PLEKHA1	-1.317907	8.0849063	-10.00473	1.0921692	1.7940066	14.3716141707276
SAMD3	-1.1762	7.6830197	-9.920058	1.3147265	2.0695987	14.1974188178879
Septin 7	-1.116994	7.8015688	-9.890129	1.4041125	2.1218948	14.1355974860117
SF3B5	0.7863093	9.0708648	9.7387840	1.9616687	2.8504556	13.8209909975084
BLOC1S1	1.4214866	9.827948	9.7187017	2.0511249	2.8700556	13.7789959350859
MLLT6	-0.875808	8.7749719	-9.66063	2.3341118	3.1493838	13.6572291902155
RFTN1	-1.245089	9.3311922	-9.642014	2.4330726	3.1697064	13.6180911401863
HS.546375	-1.742386	9.3035316	-9.397851	4.2120444	5.3043680	13.1000834543427
EDG8	-1.996146	8.6464277	-9.376385	4.4218899	5.3890000	13.0541266755503
ZBTB4	-0.966308	8.0431837	-9.321593	5.0075401	5.9120271	12.9365125738949
EOMES	-1.641656	8.7673494	-9.283147	5.4654802	6.2571467	12.8537236860829
TSPYL1	-0.826536	9.0831688	-9.259151	5.7728554	6.3997876	12.8019422666673
DDX27	-0.758319	7.4902983	-9.247468	5.9288662	6.3997876	12.7767011146506
ZDHHC12	0.9541597	7.9524052	9.1984549	6.6319767	6.9598911	12.670587
PPP2R2B	-1.210367	7.9864727	-9.135533	7.6617312	7.8232488	12.533844691319
CMTM2	1.6795529	10.777666	9.0894088	8.5196373	8.4703131	12.4332368571798
FAU	1.1523522	11.532960	9.0348589	9.6625624	9.3602977	12.3138484423304
GAPDH	0.9959100	11.296108	8.9954118	1.0586036	9.9985112	12.2272419566828
RPS21	1.1818863	7.6606179	8.9635426	1.1397890	1.0365680	12.1571058823186
SAR1A	-1.022702	8.9327627	-8.95882	1.1523520	1.0365680	12.1466994092011
CD47	-0.99664	9.3640473	-8.932283	1.2256274	1.0497190	12.0881662296856
BCLAF1	-0.59038	6.8219140	-8.925984	1.2437107	1.0497190	12.0742574617914
LEPROTL1	-1.258693	9.3649861	-8.92199	1.2553176	1.0497190	12.065435713532
C19ORF59	2.8457629	9.7092698	8.9140982	1.2785813	1.0497190	12.0479963796488
INTS9	-0.828507	8.4622216	-8.905017	1.3058971	1.0497190	12.0279182285797
EDG1	-1.460422	8.6939361	-8.847035	1.4949931	1.1766841	11.8994333499376
THEM2	0.9650538	7.2052375	8.7735301	1.7756982	1.3690996	11.7358436121205
NDUFA13	0.9656258	9.1596744	8.7554823	1.8525298	1.3801141	11.6955559665634

H2AFJ	1.43009122	8.30290384	8.75307230	1.86304455	1.38011418	11.6901723495524
MYL6	1.43130165	13.1802437	8.72070118	2.01034419	1.45191963	11.6177799021568
NCALD	-1.164674	7.70690038	-8.709789	2.06264354	1.45191963	11.5933418217938
C6ORF190	-0.954181	7.26515623	-8.707197	2.07526893	1.45191963	11.5875348226688
NMT2	-0.937232	7.66593592	-8.694581	2.13786590	1.46851952	11.5592536554426
LAPTM4B	0.81177474	6.87742284	8.67652242	2.23084260	1.50502203	11.518734060902
CD2	-1.297339	10.115741	-8.658016	2.33042782	1.54462391	11.4771593683386
HS.572219	-0.805261	9.25086311	-8.646984	2.39194505	1.55806351	11.4523525909415
S100A12	2.39059251	11.3691280	8.60888037	2.61751645	1.67609782	11.3665317639219
TGOLN2	-0.819097	9.87968234	-8.570127	2.86932828	1.79474350	11.2790304527061
HSPC171	0.74053479	7.79263519	8.55540003	2.97140422	1.79474350	11.2457215898502
UBTD1	1.17271429	7.5095395	8.55320768	2.98691489	1.79474350	11.2407602394894
HIST1H4K	1.07441147	6.80793142	8.55237571	2.99282267	1.79474350	11.2388772845489
PEX5	-0.758493	8.66875061	-8.534819	3.12031747	1.83561170	11.1991192950898
DYNLT1	1.32308272	9.07702865	8.52975125	3.15814613	1.83561170	11.1876338052216
ALDH9A1	-0.85097	11.3029327	-8.500982	3.38195821	1.93591486	11.1223643714703
HK3	1.66213901	9.3085375	8.47606535	3.58888991	2.02370538	11.0657388167129
KLRD1	-1.538263	8.0554775	-8.453878	3.78405220	2.10237488	11.0152390970694
PSCDBP	-0.912996	10.000961	-8.424363	4.06062467	2.19860308	10.9479502022811
NAT13	-0.72957	7.14049834	-8.423025	4.07364256	2.19860308	10.9448966198092
TXNDC17	1.28725641	9.26686423	8.36893845	4.63720139	2.46751364	10.821251784495
FCRL3	-1.658456	8.29793215	-8.362443	4.71005304	2.47146950	10.8063730595077
SLPI	2.68969656	7.7422675	8.31238633	5.31218875	2.74923960	10.6915159785092
ARHGEF3	-1.181652	10.2276048	-8.273444	5.83471659	2.94974107	10.6019094785287
TXN	1.78205755	9.63946084	8.27195134	5.85575914	2.94974107	10.5984706918036
COX6A1	1.00868381	9.21465680	8.26037185	6.02166696	2.99340234	10.5717815470679
RABAC1	1.03258720	9.53981130	8.25058504	6.16563445	3.02516454	10.5492091817581
PRF1	-1.925013	9.76424365	-8.231344	6.45902447	3.12013931	10.5047915501094
IRF1	-1.166104	11.8821201	-8.22718	6.52437812	3.12013931	10.4951714859886
CAST	-1.164755	8.57139388	-8.160158	7.67440109	3.62423591	10.3399950374885
FLJ14213	-1.051614	7.65916392	-8.152034	7.82723303	3.65077609	10.3211412171987
COX7A2	1.34359368	10.5327893	8.14638834	7.93526233	3.65602696	10.3080344421778
LOC642333	-1.250036	9.69342346	-8.129498	8.26766209	3.75052615	10.2687934950296
CD63	1.20426815	8.37718826	8.12596807	8.33891468	3.75052615	10.2605867769465
C1ORF55	-0.737418	7.86140838	-8.119178	8.47773106	3.76810211	10.2447970298044
LOC440280	-0.975359	8.4429965	-8.109094	8.68828422	3.81678346	10.221332769546
MAFG	0.76142005	6.82162553	8.09743031	8.93847151	3.88155694	10.194177639053
RAB32	1.37358177	9.74888726	8.08736373	9.16032465	3.93269392	10.1707243138668
ABI3	-0.867351	8.09681496	-7.986674	1.17145971	4.86348879	9.9353404758417
FYTTD1	-0.708398	7.81752611	-7.971809	1.21491388	4.98907029	9.90046784491975
STAT4	-1.148372	8.80199973	-7.960688	1.24849588	5.07184671	9.8743592947927
ARHGEF18	-0.778667	11.3393639	-7.93809	1.31969032	5.22481846	9.82124986700406
CD81	-1.331765	11.6780535	-7.935644	1.32764047	5.22481846	9.81549745720531
PTGER4	-1.196183	9.01072280	-7.886983	1.49639575	5.82823008	9.70087544978703
HMGB2	1.71231183	7.30752484	7.84870683	1.64443129	6.26015095	9.61048034903312
TRAPPC5	1.46570594	9.5426905	7.84769823	1.64852827	6.26015095	9.60809561016902
EVL	-1.384227	10.1365099	-7.84562	1.65700131	6.26015095	9.60318238848996
CD6	-1.381515	9.48296880	-7.824335	1.74640273	6.40861237	9.55281670192148
MXD4	-0.982635	9.88170546	-7.821268	1.75968453	6.40861237	9.54555433871519
ARPC5	-0.898683	12.5066765	-7.821146	1.76021896	6.40861237	9.54526326322286
CLIC3	-1.654204	8.39111284	-7.820243	1.76414951	6.40861237	9.54312519960543

GLTSCR2	-0.973401	12.7785665	-7.80452	1.83405944	6.59912056	9.50587050027389
ARMC1	-0.760377	7.98882165	-7.787132	1.91466847	6.82416743	9.46463307808051
HVCN1	-1.070609	8.47362588	-7.758941	2.05313404	7.18216706	9.39768123458186
LOC440341	-1.005616	8.32944938	-7.750972	2.09409699	7.25825543	9.37873616439507
LOC220433	-1.677079	11.5120776	-7.73868	2.15892595	7.41492931	9.34949567224047
RPA3	1.18151016	8.00236492	7.72526756	2.23200546	7.45708782	9.31756578711772
KLHDC2	-0.743772	8.47788126	-7.723318	2.24283804	7.45708782	9.31292174587853
AUTS2	-1.239968	7.34316430	-7.719786	2.26259195	7.45708782	9.30451032890127
CHCHD1	0.65017143	6.81595169	7.71937203	2.26492196	7.45708782	9.30352302316045
HIST2H2AC	1.65163349	9.58039492	7.69108444	2.42988926	7.91389795	9.23607698616379
C9ORF46	0.80678513	8.04361319	7.68553312	2.46367653	7.95535893	9.22282800497118
FCER1G	1.43553488	12.2025141	7.63408741	2.80054240	8.96648236	9.09984417234674
LOC651816	0.86787681	6.94966657	7.62325857	2.87724108	9.10435705	9.07391093703585
SUMO2	-1.26611	10.0501765	-7.621236	2.89180213	9.10435705	9.06906554051388
ATP6V0E2	-1.414559	9.02363723	-7.609091	2.98083473	9.30710217	9.03995776260861
PKM2	0.91651869	7.35228730	7.59475318	3.08955041	9.56747660	9.00556873256027
CDR2	-0.599809	7.91721365	-7.582036	3.18936032	9.79626284	8.97504330082577
S100P	2.82736229	9.66137696	7.56518157	3.32673136	1.01357992	8.93455310856288
ATP6V0E1	1.00209601	10.3816818	7.53971849	3.54580949	1.07168546	8.87330884699726
PTMA	-1.753373	10.5021749	-7.530514	3.62858123	1.08799840	8.85114725815541
TP53I11	0.71354619	7.06264623	7.50074525	3.91000225	1.16314870	8.7793997707422
CSF1R	-1.236564	10.5228151	-7.490447	4.01246326	1.18430361	8.75455041524158
LOC441743	-1.195473	8.06813884	-7.439734	4.55842157	1.33501679	8.63197683819858
GZMB	-1.943994	10.1574931	-7.42219	4.76445246	1.37405354	8.58949352291267
PLEKHF1	-1.070184	7.79725742	-7.414565	4.85693158	1.39011269	8.57101711108898
LOC116236	-0.793115	7.04809192	-7.409359	4.92113021	1.39642818	8.5583961012157
TSHZ1	-0.639566	7.17630946	-7.406806	4.95292156	1.39642818	8.55220682096047
CKLF	1.09518695	11.1704327	7.38529335	5.22929570	1.45876012	8.50001314461855
IL10RA	-0.822752	8.82378484	-7.383636	5.25122753	1.45876012	8.49598987876265
KIAA2013	0.90033981	9.11783769	7.37182134	5.41032842	1.49198691	8.4672961108332
HMGN4	-0.677088	9.67224273	-7.355224	5.64217077	1.53579107	8.42695614206009
HINT1	1.4433035	9.59066888	7.35464243	5.65047537	1.53579107	8.42554202832526
RNASE2	2.56627891	9.64343961	7.34842108	5.74010049	1.54900711	8.41041118874763
UBQLN4	-0.740078	8.29260965	-7.313426	6.27193455	1.68052260	8.32520614046027
UTP3	-1.017442	8.36713761	-7.296	6.55523012	1.73992714	8.28271810614388
GNLY	-2.26207	9.28073542	-7.294169	6.58574857	1.73992714	8.27825079574788
USPL1	-0.623342	7.20358123	-7.287344	6.70077249	1.75802211	8.26159716526566
MEX3C	-0.969369	7.775378	-7.259986	7.18276164	1.87148092	8.19478206555328
LYSMD2	-0.917873	10.4339885	-7.247197	7.42004876	1.92006467	8.16351499252181
LILRA5	1.58476031	7.73446011	7.21484136	8.05669679	2.05663516	8.0843141217288
EIF4B	-0.71874	10.7706088	-7.206939	8.22047593	2.07260197	8.06494959940311
SUCLG2	-0.760063	8.12011303	-7.205935	8.24152103	2.07260197	8.06248927966286
LTB4R	1.28443480	7.68904765	7.20391763	8.28397861	2.07260197	8.05754471530681
EAF1	-0.809993	7.57008984	-7.201334	8.33868449	2.07260197	8.05121087904008
GLRX	1.09848688	9.14232507	7.18774791	8.63245819	2.12289241	8.01789158244172
NDUFA11	0.77708223	8.68749161	7.18679750	8.65339947	2.12289241	8.01555984393708
CTSA	1.01574631	8.66386038	7.15794694	9.31432893	2.27029256	7.94472293400051
HOMER3	0.59576045	6.59900257	7.15215003	9.45321210	2.28937406	7.93047698606929
RRAGD	0.78117656	8.97605657	7.14515314	9.62365673	2.30811080	7.91327639301253
AP1S1	0.65930569	6.87179084	7.14397080	9.65276619	2.30811080	7.9103692066612
ATP6AP2	-1.020885	10.9768484	-7.140723	9.73318792	2.31270339	7.90238266848226

GNG5	0.83850644	8.67758503	7.13284066	9.93122533	2.32054855	7.88299331843535
C20ORF52	1.06323901	9.62538057	7.13259305	9.93751229	2.32054855	7.88238411551998
RPL34	0.84951588	6.97969311	7.13208311	9.95047288	2.32054855	7.88112945557199
CTSD	0.79862197	9.85114061	7.12737367	1.00709827	2.33424374	7.86954086959032
C9ORF89	0.84659152	8.57213692	7.11565288	1.03773987	2.39059831	7.84068718335792
LOC731049	1.06724168	8.748854	7.09467954	1.09495986	2.50712628	7.78901286138621
NDUFB3	1.66842179	8.61075038	7.09003676	1.10805471	2.52182572	7.77756647018755
HS.560343	-0.948667	7.57682192	-7.082127	1.13073304	2.55069018	7.7580587
RALGDS	-1.041968	10.4760238	-7.080687	1.13491064	2.55069018	7.75450780314972
CD164	-0.888409	7.80638153	-7.078602	1.14099164	2.55069018	7.74936227503339
KHDRBS1	-0.913059	10.3856905	-7.075852	1.14905803	2.55361249	7.74257884430759
CX3CR1	-0.942615	7.9383775	-7.04881	1.23155870	2.70513301	7.67580794796701
PSMB5	0.80913199	7.37975919	7.04198899	1.25330570	2.72125801	7.65895060883747
RPS10	0.82272587	12.7053389	7.02802706	1.29904426	2.79547985	7.62442893214297
CD160	-1.181972	7.16620419	-7.027056	1.30228812	2.79547985	7.62202686293231
RNASEH2B	-0.739965	7.63439042	-7.023077	1.31566685	2.80564657	7.6121827
SH2D1A	-0.772491	7.72002376	-7.021243	1.32187689	2.80564657	7.60764716392268
PRPF8	-1.064181	9.67002292	-7.014783	1.34400185	2.83157824	7.59165928965442
LSM7	0.90445716	8.20210484	7.01115809	1.35657930	2.83157824	7.58268732480335
SLAIN1	-0.808415	7.322385	-7.001856	1.38940861	2.87093894	7.55965455892702
FYN	-1.110474	9.64147796	-7.001513	1.39063479	2.87093894	7.55880483925106
SRP19	0.67203693	7.83103503	6.99869187	1.40075865	2.87612294	7.5518176
NARF	1.29748945	8.70433369	6.99452837	1.41583767	2.89137013	7.54150345531796
SNIP1	-0.665805	7.75836269	-6.974608	1.49030205	3.01088832	7.49212537547814
TC2N	-0.871488	7.05220707	-6.962973	1.53562682	3.08158151	7.46326192516504
HS.580229	-1.028933	7.68994261	-6.959546	1.54924178	3.08158151	7.45475766655647
ZNF362	-0.769036	8.50475273	-6.958954	1.55160669	3.08158151	7.45328809418985
DEF6	-0.812185	9.42329519	-6.957377	1.55791971	3.08158151	7.44937604769928
CYLD	-0.8295	8.82300965	-6.952844	1.57622513	3.10155133	7.43812140770785
NPAT	-0.695124	7.32463615	-6.946417	1.60254952	3.13701147	7.42216315383552
HS.534427	-1.638451	11.4714856	-6.941196	1.62426201	3.16312469	7.40919640356574
HIST2H2AA3	1.30949255	9.83627873	6.93237083	1.66164722	3.21933498	7.38727011514297
DNMT1	-0.972821	9.68266576	-6.924361	1.69633771	3.24999409	7.3673603662152
RPS4X	-0.992318	11.6684405	-6.922945	1.70254302	3.24999409	7.36384188551875
Septin 9	-0.868074	12.7703843	-6.922778	1.70327906	3.24999409	7.36342539064284
GSTO1	0.98458083	10.0876266	6.90675101	1.77521556	3.37023336	7.3235628726605
CSNK1G2	-0.612568	11.3115660	-6.898141	1.81513136	3.42878314	7.30213422545621
LSM10	0.75634031	8.22893365	6.89615813	1.82445021	3.42924025	7.29719918860896
RPS27L	1.37495248	8.19643819	6.89009348	1.85326094	3.46366647	7.28209937694577
RPS19BP1	0.68220043	7.38570926	6.88845955	1.86110189	3.46366647	7.27803046292104
ASXL2	-0.784518	10.0280654	-6.877369	1.91522408	3.54691990	7.25040300171843
ZFP36L1	-1.083767	11.4295933	-6.873643	1.93376031	3.56377876	7.2411198026115
SIN3A	-0.729211	8.78680638	-6.858499	2.01101373	3.68816013	7.20336364837962
BTBD15	-0.59283	7.12448465	-6.854754	2.03059483	3.70608081	7.19402379009516
RBP7	1.06577940	8.36115784	6.83857820	2.11743006	3.84598594	7.15366015381015
SP110	-1.1748	9.42909365	-6.832804	2.14933121	3.88525039	7.13924536493659
OSBPL7	-0.597037	7.2627505	-6.80328	2.32026439	4.17426613	7.06547300873928
MFNG	-0.741747	10.4223360	-6.788744	2.40943450	4.31414386	7.02911503336443
B3GNT8	1.13268916	7.15566507	6.78021649	2.46335842	4.38989063	7.00777457978945
SFRS1	-0.789628	10.1346679	-6.772783	2.51136575	4.45443183	6.9891645
COX5B	0.69541440	11.1119763	6.77043865	2.52670138	4.46069058	6.98329443313949

HS.213061	-0.65998	9.41304157	-6.762178	2.58150427	4.53624333	6.96260420606489
RPL10A	-1.524578	11.0893779	-6.756847	2.61751265	4.54705967	6.94924704325877
SCAND1	0.75998413	8.28961138	6.75592845	2.62376650	4.54705967	6.94694589424495
HS.572649	-1.410403	8.67209276	-6.748193	2.67705541	4.59993305	6.92755735182116
CBLB	-0.878395	8.56405026	-6.747967	2.67862697	4.59993305	6.92699142220454
MMP9	2.1415298	10.9974277	6.74298774	2.71352650	4.63877969	6.91450862917203
C14ORF156	1.26056423	8.31384536	6.73952388	2.73807782	4.65966577	6.9058228481675
CXCR7	-0.697837	7.10242680	-6.732582	2.78796085	4.71884534	6.88841220948834
CEBPA	0.99407973	9.67634157	6.73122337	2.79783313	4.71884534	6.88500339154511
UBE2C	1.14320359	6.72649823	6.72803093	2.82116678	4.72815458	6.87699397621741
LOC653888	0.82801052	10.5312616	6.72704912	2.82838257	4.72815458	6.87453050170654
LOC440731	1.25479006	8.12166180	6.72466761	2.84596332	4.73658565	6.86855459654994
LOC203547	-0.58541	8.79860173	-6.718381	2.89291047	4.79360340	6.85277557669248
VNN1	1.4826218	7.45897830	6.71296517	2.93397995	4.84042632	6.83918033627343
LCK	-0.626785	7.37868919	-6.700037	3.03443751	4.98439344	6.80671077533315
BTN3A3	-0.749786	7.64177626	-6.697128	3.05751769	4.99365711	6.79940259667652
ELF1	-0.746256	10.7632063	-6.696	3.06651257	4.99365711	6.79656933854073
LOC729776	0.71904712	7.28350776	6.69243648	3.09511771	5.01860717	6.7876139787616
C3AR1	1.22511402	7.34682988	6.68559763	3.15077182	5.08701536	6.77042483659149
PYCARD	0.80443352	11.3945557	6.67383606	3.24887527	5.22308543	6.74085039752739
UNC84B	-0.917	12.5215531	-6.646772	3.48657132	5.58146883	6.6727389536324
HS.571245	0.72047922	7.17334419	6.64029541	3.54602470	5.65269255	6.65642794625069
SH3YL1	-0.710684	7.34949930	-6.637021	3.57647913	5.67728493	6.64817826550237
NOLA3	1.05163154	11.3066607	6.62957487	3.64671053	5.75472930	6.62941792266836
LOC642161	-0.605835	6.9167225	-6.628629	3.65573063	5.75472930	6.62703464630552
KIAA0999	-0.607537	8.88301684	-6.625282	3.68784021	5.76110874	6.61859810851029
GBA	0.8983259	8.04132290	6.62502851	3.69028141	5.76110874	6.61795970736246
EIF4A2	-0.691499	11.1839303	-6.619369	3.74525913	5.82287613	6.60369301783405
MYD88	0.87810050	10.0509108	6.61620821	3.77632598	5.83958053	6.59572339313624
CNIH4	1.1055488	8.85522184	6.61513668	3.78691690	5.83958053	6.59302143214922
LOC147804	-0.778443	7.14863726	-6.60664	3.87197302	5.93419765	6.57159179713696
YWHAQ	-0.928093	11.1421959	-6.605878	3.87968983	5.93419765	6.56967088564645
TAF4	-0.856516	7.95071590	-6.602951	3.90950603	5.95569104	6.56228455689218
CCDC109A	0.64304623	7.54448776	6.59171588	4.02609353	6.10427253	6.53393277142266
FGFBP2	-1.534414	9.01904803	-6.590459	4.03935450	6.10427253	6.53076004102755
G3BP1	-1.020432	8.34149484	-6.583552	4.11302780	6.19084424	6.51332074666749
LPIN1	-0.770808	8.38043661	-6.577094	4.18314020	6.27139035	6.49701170349192
SCAP	-0.757703	9.88993011	-6.566401	4.30190212	6.39865600	6.46999923274659
ACAA1	1.03969654	8.00236240	6.56416119	4.32721300	6.41106303	6.4643386150632
PPARBP	-0.762611	7.94350357	-6.557799	4.39993335	6.46807324	6.44825731187207
EIF3A	-0.996734	8.85803669	-6.556212	4.41826471	6.46984655	6.4442454
C2ORF29	-0.628165	9.66872203	-6.547936	4.51511731	6.58614409	6.42332108350002
C14ORF2	0.97056333	7.56059103	6.53293623	4.69619435	6.79778630	6.38537622808015
COX7B	1.22680015	7.63739169	6.53053725	4.72583093	6.81457606	6.37930534548184
CKAP4	1.35926063	9.71573415	6.52650714	4.77604613	6.86079934	6.36910533828116
TXK	-0.906874	7.35121342	-6.524503	4.80122387	6.87084235	6.3640313
EP400	-0.675112	7.93218190	-6.518534	4.87699592	6.95293985	6.34892002150951
RPS28	-1.359993	13.133765	-6.50095	5.10734615	7.25396759	6.30438095081916
MYL9	1.97506884	8.03750776	6.49489917	5.18913573	7.34252989	6.28904791900723
DOCK10	-0.973286	8.9670705	-6.489397	5.26466040	7.41006228	6.27510223951802
CA5B	-0.858565	7.78843392	-6.488572	5.27608987	7.41006228	6.27300921872475

GLOD4	-0.648547	7.91583015	-6.473193	5.49364577	7.68703471	6.23401001397743
INADL	-0.754112	7.415792	-6.470346	5.53490506	7.71618868	6.22678818368091
MRPL33	0.76889772	8.20588469	6.45925741	5.69864362	7.91524839	6.19864877038206
EEF2	-0.731402	12.4664998	-6.446385	5.89488696	8.15783258	6.16596853753159
ZMIZ2	-0.595965	7.12449707	-6.443015	5.94739546	8.20045987	6.15740859390537
HS.568928	-0.655706	8.4224395	-6.440303	5.98998873	8.22915543	6.15052030815434
MRPL54	0.67850123	8.2746725	6.40910585	6.50279218	8.86915121	6.07122772881063
PRKRA	-0.630261	7.90769419	-6.407079	6.53761688	8.88457436	6.06607173661528
METTL7B	1.28209645	6.65036088	6.40562346	6.56273061	8.88673700	6.06237050783698
CD7	-1.385792	9.97494053	-6.394266	6.76212655	9.12404075	6.03347634588322
ZNF313	-0.624321	9.97796623	-6.383795	6.95140837	9.33018453	6.00682478552669
DRAM	1.02274047	7.49990865	6.38309286	6.96429867	9.33018453	6.00503624267974
ITGA2B	1.50093663	8.11462026	6.36222700	7.35839928	9.82333303	5.95189342986169
TLR1	-1.038011	9.91167373	-6.347265	7.65484911	0.00010183	5.91376018668087
FLI1	-0.984058	7.59933542	-6.333679	7.93451116	0.00010518	5.87911550211171
SGSH	0.65549290	8.41030234	6.32249552	8.17247108	0.00010795	5.85058444486107
ITPR3	-0.910547	8.20508296	-6.320576	8.21404336	0.00010812	5.84568529196219
RETN	2.19368777	7.16475507	6.31567636	8.32110756	0.00010896	5.83318131543553
SP4	-0.889812	7.65181719	-6.315047	8.33496073	0.00010896	5.83157516871296
SEC11A	1.01036072	9.4405845	6.31356223	8.36774321	0.00010901	5.82778494338069
C17ORF61	1.05076104	8.68416792	6.30428246	8.57559885	0.00011133	5.80409309574014
MBNL2	-0.737209	7.22592923	-6.295446	8.77839144	0.00011282	5.78152499167226
KIAA0182	-0.647647	7.42596703	-6.294947	8.78997442	0.00011282	5.78025173627219
SH3BP5L	0.70574490	8.563458	6.29409033	8.80992623	0.00011282	5.77806246458623
EXOSC10	-0.956219	8.77878588	-6.286655	8.98495760	0.00011397	5.75906630959374
RBBP7	-0.853263	7.89549107	-6.286438	8.99011408	0.00011397	5.75851230059298
FAM43A	-0.762508	8.03435380	-6.282984	9.07266154	0.00011463	5.74968641792885
PERLD1	-0.648212	7.31919934	-6.281625	9.10536369	0.00011466	5.74621211225765
SGK	-0.993877	10.4588415	-6.280078	9.14272032	0.00011475	5.74225853459829
ZMIZ1	-0.901426	10.1936293	-6.264702	9.52256099	0.00011912	5.70295123130749
AGTRAP	0.82247148	8.93048653	6.25325396	9.81574366	0.00012238	5.67366856264768
PGD	1.13541261	10.7135296	6.22511950	1.05756909	0.00013099	5.60165570141208
LOC730316	-0.794804	10.2999723	-6.223532	1.06203138	0.00013112	5.59758950482948
VPS37C	-0.728265	10.0316956	-6.219185	1.07434542	0.00013221	5.58645644603417
CD58	1.04860381	8.09035788	6.21794517	1.07788409	0.00013221	5.58328074090553
TMEM66	-0.801128	12.2037011	-6.216582	1.08178900	0.00013226	5.57978843030735
MALT1	-0.808292	7.33217423	-6.213823	1.08973510	0.00013266	5.57272065362157
PTTG1	0.75490062	6.85119061	6.21301042	1.09208645	0.00013266	5.57063908698801
C17ORF62	0.91371422	9.76966561	6.21029441	1.09998393	0.00013319	5.56368035927739
GPR114	-0.850958	7.82234330	-6.208176	1.10618480	0.00013351	5.55825147406878
ARL2BP	-0.643624	9.08313511	-6.199584	1.13169411	0.00013547	5.5362333395139
GRK6	0.62317669	7.92274473	6.19823857	1.13574332	0.00013547	5.53278398524028
LOC374395	-0.662926	11.1271743	-6.197907	1.13674349	0.00013547	5.53193386909704
U2AF2	-0.765465	10.204869	-6.191227	1.15707995	0.00013707	5.51480879222553
MRFAP1L1	-0.782291	9.21541053	-6.191133	1.15736824	0.00013707	5.51456819087678
CYFIP2	-0.813063	11.1277722	-6.173695	1.21222364	0.00014311	5.46984450513648
NCF4	0.81209629	7.37611211	6.17055351	1.22238267	0.00014386	5.46178428008542
CLIC1	0.85519490	11.818079	6.15499755	1.27397068	0.00014834	5.4218605226386
CD5	-0.953126	8.058083	-6.153931	1.27758796	0.00014834	5.41912205198737
HS.436134	-0.906546	7.66600980	-6.153651	1.27854021	0.00014834	5.41840243388823
KIAA0194	-0.592151	7.53788919	-6.153215	1.28002204	0.00014834	5.41728368161674

C19ORF2	-0.782094	9.25613511	-6.148483	1.29622511	0.00014936	5.40513441940124
LGALS1	1.0060825€	11.3880584	6.14750992	1.29958213	0.00014936	5.40263627413521
HP	2.02686522	7.22167461	6.14718262	1.30071350	0.00014936	5.40179581097872
ZFP36L2	-1.178779	9.16323680	-6.143334	1.31409051	0.00015044	5.39191343792384
PHF23	-0.597463	8.14840138	-6.134464	1.34545677	0.00015356	5.36913015484155
COMTD1	0.65125887	6.90716334	6.12465480	1.38102778	0.00015668	5.34392645051207
MOSC1	1.30039990	8.94411276	6.09693279	1.48678610	0.00016767	5.2726547282557
HLA-DMB	-1.236716	11.0766354	-6.091667	1.50778273	0.00016955	5.25910946644307
LOC728481	-1.129968	12.4396309	-6.080798	1.55207365	0.00017368	5.23114458630614
KLF12	-0.594239	6.88908246	-6.080366	1.55386454	0.00017368	5.23003068217368
ATF6	0.88195563	8.77648307	6.07595812	1.57222033	0.00017521	5.21868710688809
CTSO	-0.738328	7.94702461	-6.065146	1.61818410	0.00017826	5.19085308689136
LRFN3	-0.853972	7.39871292	-6.065091	1.61842080	0.00017826	5.19071180097432
GBP4	-1.055143	8.02264084	-6.054877	1.66309726	0.00018265	5.16440843353488
CLPTM1L	0.65010558	8.73465765	6.04523382	1.70641993	0.00018686	5.13956817674141
ARG1	2.42873748	7.52221523	6.04157088	1.72317382	0.00018788	5.1301305349131
TXNIP	-1.001463	12.6251533	-6.041023	1.72569225	0.00018788	5.12871981259199
MAN2B2	-0.865551	9.32951873	-6.035301	1.75223904	0.00019022	5.11397332516557
SFRS5	-0.719856	10.8588643	-6.027967	1.78686567	0.00019305	5.09507069306908
RILPL2	0.64220218	8.77232111	6.02767240	1.78827169	0.00019305	5.09431091109664
RFXANK	0.66995138	8.8223885	6.02362772	1.80767943	0.00019457	5.08388391912361
TRIT1	-0.675952	7.32924253	-6.013915	1.85516047	0.00019885	5.05883883207751
CARD11	-1.057166	8.3482125	-6.013338	1.85801871	0.00019885	5.05735169655117
PDCD4	-0.658538	7.45883773	-6.011145	1.86892980	0.00019945	5.05169560212572
IFP38	-0.95905	9.42398007	-6.000955	1.92048488	0.00020323	5.02540953609674
MEF2D	-0.64783	7.96100053	-5.989951	1.97776973	0.00020847	4.99701702131267
ZMYM6	-0.64073	8.92019692	-5.989086	1.98234571	0.00020847	4.99478457361167
CFL1	-0.608108	12.7402762	-5.988306	1.98648258	0.00020847	4.99277078323151
CTGLF3	-0.917976	9.09988230	-5.982948	2.01512675	0.00021030	4.9789410237321
RNF44	-0.649996	8.95986157	-5.981308	2.02397472	0.00021064	4.97470882544923
BMS1	-0.644516	7.93156015	-5.979361	2.03453603	0.00021116	4.96968124405216
RPUSD2	-0.658511	8.25084111	-5.960799	2.13802964	0.00022130	4.92175108397948
PPP1R2	-0.710859	8.34163957	-5.959494	2.14550506	0.00022137	4.91837941268506
TMEM126A	0.75126740	7.42228742	5.95863572	2.15043559	0.00022137	4.9161619963792
FLJ21438	-0.885282	10.5240798	-5.944123	2.23554720	0.00022888	4.87866558832841
CAMKK2	0.59182704	6.54459596	5.93554236	2.28746334	0.00023293	4.85648826975529
GYG1	1.50210741	10.2733972	5.93389140	2.29759115	0.00023334	4.85222062753113
ZNF329	-0.616364	7.07191107	-5.917527	2.40046293	0.00024248	4.80990849701621
ZNHIT1	0.77078433	9.22993957	5.91438879	2.42071563	0.00024359	4.80179233912939
TNFAIP6	1.84758730	9.30061234	5.91383637	2.42429865	0.00024359	4.80036353238091
PCSK7	-0.586516	8.26411746	-5.909481	2.45273396	0.00024579	4.78909866628072
ELP3	-0.711409	8.4732895	-5.906335	2.47348947	0.00024721	4.7809584
ZDHHC8	-0.749207	9.38773611	-5.903259	2.49394700	0.00024860	4.77300147752893
NOL5A	-0.695063	7.85403353	-5.8982	2.52796929	0.00025067	4.75991205426255
RGS14	0.61272344	7.59973534	5.88644178	2.60887060	0.00025734	4.72948109509368
GZMK	-1.247192	8.71233853	-5.879834	2.65547521	0.00026126	4.71237635671891
RIN3	0.83314541	7.43943526	5.87408833	2.69668584	0.00026415	4.69749955906031
IRF8	-0.851605	9.79184026	-5.873787	2.69886567	0.00026415	4.69671899619401
JUNB	0.90930563	8.46270546	5.86404439	2.77028050	0.00027029	4.6714891616604
LOC728499	-1.134846	9.06031888	-5.863291	2.77588214	0.00027029	4.6695377735578
LRPAP1	0.92799124	7.26904726	5.85410761	2.84508647	0.00027631	4.64574940192431

NDUFV2	0.87752634	8.93011088	5.84693199	2.90037146	0.00028024	4.62715781537096
RAB22A	-0.660483	8.63729788	-5.842748	2.93310503	0.00028168	4.61631628343752
FTHL11	-1.038081	12.3307209	-5.841867	2.94004924	0.00028168	4.61403188727503
OSCAR	1.10727405	9.07082265	5.84122500	2.94511503	0.00028168	4.61236882414183
C5ORF32	1.40340047	9.01506530	5.83910049	2.96194890	0.00028258	4.60686286141085
CHCHD9	0.71006558	9.70204388	5.83810102	2.96990197	0.00028262	4.60427247275101
MRPS25	-0.620544	7.14652307	-5.83443	2.99929665	0.00028470	4.59475819701595
LPPR2	0.82054750	8.39313515	5.83100627	3.02697999	0.00028661	4.5858827193613
NARS	-0.809788	9.64856223	-5.825742	3.07004981	0.00028924	4.57223434492395
E4F1	-0.830792	8.82427069	-5.822834	3.09409961	0.00029029	4.56469629667233
LOC646630	0.66840236	11.0533851	5.81955131	3.12148397	0.00029029	4.55618409697693
POLE4	0.69470201	9.27408157	5.81884967	3.12736848	0.00029029	4.5543646991178
DIMT1L	-0.630282	8.25124946	-5.809593	3.20605542	0.00029542	4.53035958509242
NUP62	-0.838062	9.4680305	-5.799542	3.29376585	0.00030203	4.50428651487789
SH3KBP1	-0.780538	7.10068753	-5.79372	3.34567630	0.00030605	4.48918060782661
SERPINB1	0.92241562	10.3771621	5.78938764	3.38483730	0.00030814	4.47793910756768
HECA	-0.910746	9.46404534	-5.782833	3.44496572	0.00031194	4.46092944606076
HS.534439	-1.357229	8.19200607	-5.782144	3.45135438	0.00031194	4.45913964443598
SSR4	0.61924744	10.8174872	5.77752915	3.49441469	0.00031508	4.44716192980036
ARHGAP17	-0.722381	9.02294988	-5.773387	3.53353140	0.00031718	4.43640845169701
STARD10	0.74878722	7.66641084	5.77327904	3.53455321	0.00031718	4.43612914759524
HS.550293	-0.968969	10.6422217	-5.768822	3.57714531	0.00031969	4.42455815547594
LPXN	-0.831516	10.7386731	-5.765805	3.60627468	0.00032116	4.41672366173106
CKS2	0.68165669	6.81367507	5.76512362	3.61288174	0.00032116	4.41495546521977
PGLYRP1	1.63671772	10.3998112	5.75319706	3.73059570	0.00032981	4.38398340409361
EXOSC4	0.99841759	7.06919369	5.75261571	3.73643148	0.00032981	4.38247347513259
HIST1H4C	1.30050888	11.7054221	5.74977322	3.76509827	0.00033131	4.37509042416641
NLRC4	1.09229265	7.22058726	5.74920023	3.77090372	0.00033131	4.37360209250373
KLHL3	-0.613261	7.04975392	-5.742078	3.84382190	0.00033624	4.3551009811583
DPH3	0.67521209	7.28592034	5.74198371	3.84479884	0.00033624	4.35485549709536
C11ORF56	0.67157486	8.00262530	5.73782667	3.88802713	0.00033923	4.3440552024135
C9ORF16	1.11966620	7.37720984	5.73691460	3.89757706	0.00033928	4.34168541756781
MRPS6	-0.629872	10.5235858	-5.735867	3.90857067	0.00033946	4.33896457142417
PLSCR3	-0.835753	10.7170730	-5.730279	3.96777063	0.00034381	4.32444331523427
FTHL8	-0.740516	13.2956790	-5.727508	3.99746558	0.00034559	4.31724080629618
RNF165	-0.944026	7.12872769	-5.723543	4.04033728	0.00034850	4.30693613508113
HNRPUL1	-0.620223	10.0808876	-5.716939	4.11276707	0.00035394	4.28977284689295
PIGU	-0.597402	7.65123980	-5.707617	4.21725902	0.00036210	4.2655373888817
ST3GAL4	0.86524033	7.31809846	5.70602352	4.23538728	0.00036284	4.26139401829286
MAPBPIP	0.63041756	8.64811619	5.70352236	4.26399863	0.00036384	4.25489060032146
EDG6	0.84219713	11.0227346	5.69568847	4.35488000	0.00037055	4.23451890279128
IRAK3	1.71620697	8.33243646	5.69249955	4.39243204	0.00037291	4.22622524037915
SLC19A1	0.61224008	7.66424623	5.6803213	4.53887080	0.00038448	4.19454695847135
NKTR	-0.828424	9.19201823	-5.677992	4.56743941	0.00038603	4.1884862071772
LOC388789	0.59781348	8.787421	5.67036784	4.66220616	0.00039316	4.16864983774235
EML4	-0.859324	9.07179319	-5.668576	4.68476285	0.00039418	4.16398776439564
SMAD4	-0.650943	8.80841988	-5.66055	4.78716702	0.00040190	4.1431011797509
SLAMF6	-0.679515	7.58241146	-5.659552	4.80006129	0.00040201	4.14050297615931
TPST1	1.62526520	7.54758026	5.65880770	4.80969953	0.00040201	4.13856542081049
TAF1C	-0.728138	8.97741969	-5.65568	4.85041234	0.00040452	4.13042364425912
LOC645058	0.68344973	9.90929411	5.65241811	4.89323266	0.00040604	4.12193384819707

ASCC2	-1.735846	12.1341795	-5.652312	4.89462668	0.00040604	4.12165871205995
LFNG	-0.884427	11.2711658	-5.65184	4.90086107	0.00040604	4.12042919961175
NELF	-0.663684	7.36143134	-5.638964	5.07393917	0.00041854	4.0869062
SH2D1B	-1.002947	7.04893753	-5.637453	5.09464538	0.00041933	4.08297252007171
IRX3	0.61563011	6.64532903	5.63564484	5.11954142	0.00041979	4.07826400501774
PTGES3	-0.886234	9.70752869	-5.635434	5.12244665	0.00041979	4.07771604154664
IMP3	-0.723888	10.1290903	-5.620525	5.33256831	0.00043607	4.03888703107577
JMJD1A	-0.586349	7.68324688	-5.618524	5.36142218	0.00043692	4.03367494114705
TPST2	0.73276752	9.78983665	5.61819713	5.36615593	0.00043692	4.03282252839454
SPTAN1	-0.782927	8.24270919	-5.613447	5.43534287	0.00044160	4.02044908802582
SBDS	-0.619091	7.46867426	-5.604626	5.56623577	0.00044934	3.99746529518229
LOC653610	0.94493934	7.33275384	5.60194050	5.60670185	0.00045164	3.99046909596334
HLA-DQA1	-1.626443	9.34291765	-5.597429	5.67536123	0.00045585	3.97871333236262
ADORA2A	0.81897962	7.92834473	5.58501923	5.86863436	0.00046874	3.94637003136748
C1ORF63	-0.844146	9.18354646	-5.58042	5.94193960	0.00047351	3.93438077686274
MTHFS	0.80656863	8.77150557	5.57970580	5.95340082	0.00047351	3.93251965522673
RGS19	1.12013293	9.48849423	5.57842098	5.97408249	0.00047416	3.92917033118865
SRGN	0.81469311	13.5757481	5.57585278	6.01563967	0.00047645	3.92247522705423
PEA15	-0.857512	9.13183819	-5.571508	6.08661229	0.00048107	3.91114742171507
HNRPDL	-0.653342	7.68708880	-5.563436	6.22070544	0.00048973	3.89010130820021
COX17	0.81911448	9.16698261	5.56217106	6.24198858	0.00048973	3.88680268748214
NOV	-0.959895	7.82813084	-5.561911	6.24637681	0.00048973	3.88612396685582
DDIT3	0.68974990	6.83111696	5.56181223	6.24803916	0.00048973	3.88586697834034
SMARCA4	-0.658371	8.62876438	-5.555733	6.35145090	0.00049680	3.87001324193515
NELL2	-1.177574	7.97218	-5.554087	6.37974982	0.00049798	3.86571982383183
CTSW	-1.407483	9.03121519	-5.544724	6.54312260	0.00050655	3.84130005691989
NQO2	1.26626552	7.55816588	5.54224860	6.58701703	0.00050891	3.83484299883011
EGLN2	0.73841870	8.73132142	5.54037534	6.62043138	0.00051044	3.8299564054121
NEU1	0.61005077	7.62394065	5.53638041	6.69226206	0.00051493	3.81953474060475
CCDC53	0.71089423	8.22877657	5.53234328	6.76564901	0.00051952	3.80900225141932
OSBPL5	-0.713717	7.54317542	-5.525179	6.89788127	0.00052755	3.79030968666348
LY96	1.70428202	9.42326619	5.52333408	6.93235371	0.00052909	3.78549552844844
ATP8B2	-0.724502	7.745875	-5.51763	7.04003101	0.00053515	3.77061097069025
CAMP	2.2972614	10.0706166	5.50238158	7.33622923	0.00055211	3.73081286115833
PIK3IP1	-1.002735	9.69356438	-5.494786	7.48842337	0.00056115	3.71098468714138
KLHDC8B	1.01279169	8.46831226	5.49440045	7.49623012	0.00056115	3.70997851044199
MTX1	0.63551220	8.74056338	5.49408945	7.50253542	0.00056115	3.70916661519176
PRICKLE4	0.78819579	8.63899961	5.49274545	7.52984560	0.00056115	3.70565790542956
SLC22A4	1.1062398	8.03256096	5.49097347	7.56600568	0.00056240	3.70103175644925
SF3B3	-0.738006	8.14760607	-5.490428	7.57716884	0.00056240	3.6996080608426
ARPC1A	0.72726402	8.13607976	5.48469649	7.69550902	0.00056703	3.68464331398654
DPM3	0.85761072	7.5319325	5.48383141	7.71353103	0.00056703	3.68238456323105
LOC400464	-0.677157	7.2734755	-5.483485	7.72076346	0.00056703	3.68147958660478
SRPK1	1.10395499	9.20295996	5.4832486	7.72569573	0.00056703	3.68086290932418
TJAP1	-0.736976	8.60650634	-5.483062	7.72959462	0.00056703	3.68037571344141
PVRIG	-1.146896	8.47641246	-5.478164	7.83266472	0.00057348	3.66758474845992
NME1-NME2	0.68449001	8.69583992	5.47369833	7.92782078	0.00057932	3.65592458980843
DDEF1	0.98083827	10.7173451	5.46829296	8.04457123	0.00058599	3.64180819572577
RPS9	0.62729131	11.5705397	5.46803817	8.05011693	0.00058599	3.64114277289568
SSNA1	0.61272130	6.98420957	5.46538748	8.10804012	0.00058794	3.63421991233491
STX10	0.65511534	9.88273723	5.46164188	8.19060511	0.00059166	3.62443700407273

NDUFA1	1.2193337	10.682963	5.4570182	8.2936921	0.0005979	3.61236012282945
MYC	-0.721567	8.9830099	-5.456032	8.3158519	0.0005981	3.60978367312208
HS.554507	-0.780842	7.1483226	-5.436554	8.7658553	0.0006213	3.55889904023158
LIN7A	0.8801014	6.9843134	5.4365431	8.7661233	0.0006213	3.55886951414281
TMCO3	0.9594427	7.7169212	5.4245652	9.0549528	0.0006382	3.52757054295981
PRRC1	-0.726631	7.6901275	-5.419519	9.1794860	0.0006446	3.51438267925547
BTG1	-0.62379	12.827116	-5.417627	9.2266200	0.0006467	3.50943793732328
MFN2	1.0086010	8.3639981	5.4132296	9.3371090	0.0006532	3.49794512355462
HEATR1	-0.784478	7.7177722	-5.403211	9.5938297	0.0006652	3.4717588258972
LOC387820	-0.643507	10.575634	-5.403034	9.5984225	0.0006652	3.4712967572462
MAP4K1	-0.853727	8.7965269	-5.402413	9.6145859	0.0006652	3.46967236911481
BST1	0.8095640	7.8319457	5.4013056	9.6434575	0.0006660	3.46677762476263
SERTAD2	-0.721778	9.3048059	-5.397311	9.7483292	0.0006708	3.45633540985528
AHNAK	-0.982264	9.9445269	-5.396604	9.7670061	0.0006709	3.45448751952893
FAIM3	-1.377496	10.736934	-5.389565	9.9549869	0.0006825	3.43608334907192
VPS26	-0.612059	7.3982763	-5.381419	1.0177029	0.0006940	3.41478732014065
HYAL3	0.7997681	7.0393683	5.3768957	1.0302493	0.0007013	3.40295895559479
C1QB	0.7352564	6.6215510	5.3718436	1.0444452	0.0007084	3.38974801693792
UGCG	0.9001410	8.6943985	5.3692002	1.0519510	0.0007100	3.38283546569008
DOK3	0.5890505	7.2741371	5.3687743	1.0531654	0.0007100	3.3817217146485
RIT1	0.6432189	6.8553954	5.3686859	1.0534175	0.0007100	3.38149065524434
FLJ10081	-0.772712	8.3375728	-5.367841	1.0558304	0.0007100	3.37928198177852
GNB2	0.7964368	8.4076792	5.3676740	1.0563091	0.0007100	3.3788444496773
ACTR3	-0.736063	10.532983	-5.366165	1.0606354	0.0007117	3.37489880583073
CCNB2	0.8621227	6.5767448	5.3593029	1.0805389	0.0007225	3.35695184349185
SYNJ2BP	-0.777831	8.1520528	-5.351577	1.1033975	0.0007339	3.33674405045157
RARRES3	-0.925778	10.465514	-5.346896	1.1174823	0.0007419	3.32450032413481
SFT2D1	0.6060143	10.412609	5.3439116	1.1265554	0.0007453	3.3166946931525
KLHL22	-0.67123	8.7138507	-5.341166	1.1349692	0.0007496	3.30951236336616
LOC643287	-0.957667	8.9306022	-5.336496	1.1494234	0.0007578	3.29729716087543
ENTPD1	0.7507352	7.7095027	5.3327440	1.1611726	0.0007642	3.28748081261428
TRIM25	1.1308601	8.4225751	5.3148280	1.2189558	0.0007967	3.24060649926569
TLR5	1.5254272	8.9898829	5.3119522	1.2284962	0.0008015	3.23308177180669
NDUFB6	0.6193114	9.2713902	5.3072216	1.2443538	0.0008105	3.22070313408746
LOC442535	-0.664654	7.0142169	-5.303538	1.2568450	0.0008158	3.21106305758745
NUDT22	0.5906528	7.412002	5.2929652	1.2933974	0.0008352	3.18339517578827
MRPS15	0.6041503	7.8656556	5.2885683	1.3089113	0.0008424	3.1718880792384
TMEM131	-0.743675	9.6949258	-5.287869	1.3113964	0.0008425	3.17005751884185
FAM10A4	-1.162924	10.156465	-5.282211	1.3316739	0.0008527	3.15524918938978
RNF213	-3.179905	11.865385	-5.278046	1.3468002	0.0008609	3.14434904250886
GNG8	0.8023093	6.6392219	5.2595950	1.4159222	0.0009000	3.09605043386155
AGPAT1	0.6887068	7.6655489	5.2591671	1.4175668	0.0009000	3.09493024971311
ZSCAN18	-0.772342	7.8317896	-5.256273	1.4287403	0.0009041	3.08735391484789
NDUFB2	0.8064117	9.2701691	5.2546775	1.4349378	0.0009050	3.08317716878149
LOC137107	-0.594935	7.0031829	-5.253958	1.4377417	0.0009052	3.08129347423463
TNFSF10	0.8686841	10.013519	5.2415801	1.4868451	0.0009320	3.04888800080777
TDRD9	1.1632304	6.7454937	5.2413798	1.4876532	0.0009320	3.04836370403294
ROPN1L	1.0679740	8.5333231	5.2382068	1.5005164	0.0009358	3.04005630019151
RFX5	-0.601476	8.6111785	-5.238052	1.5011468	0.0009358	3.0396510279581
RAG1AP1	0.6398184	7.9333749	5.2351116	1.5131722	0.0009409	3.03195218418027
LOC400455	-0.650581	8.9627395	-5.226191	1.5502486	0.0009585	3.00859533496519

PGS1	1.25679573	9.38301126	5.22486657	1.55583088	0.00096024	3.0051272777203
HNRPH3	-0.731381	8.38425076	-5.224343	1.55804492	0.00096024	3.00375520849465
YY1	-0.65045	10.3719396	-5.222584	1.56550004	0.00096326	2.99914949032668
GZMA	-1.067641	9.09281707	-5.221035	1.57209524	0.00096575	2.99509330469316
ATP5EP2	0.77676065	13.097086	5.21899474	1.58082223	0.00096796	2.98975213469136
NDUFAF1	0.70928677	6.50448330	5.21464943	1.59957562	0.00097470	2.97837373692143
RPLP1	-1.291127	11.9411293	-5.212864	1.60734769	0.00097786	2.97369723145156
SHKBP1	0.81767676	9.29927196	5.20535529	1.64043990	0.00099320	2.95403570098328
ANKRD9	0.62674656	6.62329515	5.20364010	1.64809491	0.00099624	2.94954411256377
ZBTB40	-0.792884	8.28701023	-5.200462	1.66237402	0.00100326	2.94122138449148
LPAR5	-0.841385	8.42116630	-5.199438	1.66700110	0.00100445	2.93853979334309
HS.135282	-0.610679	7.05335430	-5.196995	1.67809207	0.00100792	2.93214234010352
ARHGAP1	-0.595379	9.65063888	-5.189416	1.71297567	0.00102561	2.91229348622814
SFRS11	-0.827661	7.66388823	-5.185692	1.73038239	0.00103414	2.90253992395964
ADNP	-0.665485	8.02136238	-5.185199	1.73269805	0.00103414	2.90124979911853
CSTA	0.93843659	7.5291985	5.18245463	1.74556644	0.00104023	2.89406198132075
LEF1	-1.04031	9.32712419	-5.174833	1.78215647	0.00105980	2.87409964654544
ECHDC3	1.40694218	7.45560323	5.16361500	1.83727280	0.00108710	2.84471882606782
SCAMP3	-0.606478	8.94891165	-5.16333	1.83869583	0.00108710	2.8439719969912
PSCD1	-0.588428	11.5792520	-5.159522	1.85780837	0.00109497	2.8339971494991
KIAA0748	-0.965313	7.8285505	-5.154268	1.88450207	0.00110382	2.82023638356191
PPP1R16B	-0.726706	7.84015380	-5.152648	1.89281090	0.00110697	2.81599295235841
HCP5	-0.686995	11.4087475	-5.150692	1.90289140	0.00111036	2.81086968803677
LOC255809	0.58594402	7.86923669	5.13866511	1.96606892	0.00114273	2.77936690276518
CKS1B	0.70245458	7.14684653	5.13519004	1.98471234	0.00115003	2.77026415837983
DHRS3	-0.613309	7.13994192	-5.130316	2.01115747	0.00115860	2.75749804204261
HCFC1	-0.681687	9.78644092	-5.127158	2.02848681	0.00116371	2.74922335968359
AGPAT2	0.68069837	6.97420365	5.12690401	2.02988451	0.00116371	2.74855905499352
PUF60	-0.686207	10.053649	-5.119253	2.07250758	0.00118635	2.72851796499682
POLR2I	0.63980815	7.86299226	5.10627401	2.14688019	0.00122336	2.6945174830831
S100A8	1.11246480	13.6913873	5.10345129	2.16340550	0.00122907	2.68712304089968
RTN1	-0.838346	8.154784	-5.098789	2.19098118	0.00123959	2.67490906992832
TMEM43	-0.654337	10.0573928	-5.098657	2.19176350	0.00123959	2.67456478365112
MYL6B	0.74889627	7.39974484	5.09615876	2.20669143	0.00124431	2.6680192786004
NSUN2	-0.715137	9.06208846	-5.0948	2.21484965	0.00124704	2.66446082273483
SF3A3	-0.726704	9.12731111	-5.093726	2.22132592	0.00124883	2.6616453
HLA-DPA1	-1.367773	11.2045038	-5.087266	2.26065101	0.00126529	2.64472378965592
RNASE3	2.26465813	7.5987065	5.07992318	2.30620324	0.00128691	2.62548753355661
RPA2	-0.659304	9.90019776	-5.079396	2.30950615	0.00128691	2.62410758554999
C1ORF78	0.72566037	6.94640511	5.07758311	2.32091220	0.00129137	2.61935731632145
GLT25D1	0.70387266	9.36391853	5.06725393	2.38696978	0.00132228	2.5922981553815
CCNG1	-0.756456	8.53590007	-5.0627	2.41668792	0.00133678	2.58036852294285
LOC642817	-0.840532	9.22414742	-5.050299	2.49950791	0.00137856	2.54788227249649
STAT2	-0.827603	10.8518154	-5.048508	2.51170241	0.00138326	2.54319026865813
SIRT1	-0.60614	7.66212115	-5.041322	2.56123061	0.00140644	2.52436536456329
RDH11	-0.593571	9.9309935	-5.033999	2.61271306	0.00142848	2.50518042778037
ITFG2	-0.602408	7.87310411	-5.032799	2.62124582	0.00143073	2.50203732932487
PTGDS	-1.041154	7.39464126	-5.032356	2.62440023	0.00143073	2.50087797364795
RPS6KA5	-0.882097	9.52056811	-5.031078	2.63353345	0.00143364	2.49752906485977
LOC648210	-0.964674	10.2357112	-5.029737	2.64314792	0.00143680	2.49401624029088
ARPC1B	0.72237368	9.05865276	5.02267020	2.69439654	0.00146046	2.47550501686584

OSBPL11	-0.726807	7.71554373	-5.017834	2.73004457	0.00147760	2.46283568915624
ATP5J2	0.64795573	9.61727688	5.01681090	2.73764388	0.00147965	2.46015633303466
TK1	0.78607799	6.92032665	5.01355427	2.76198071	0.00148660	2.45162556465276
LOC653080	-0.65897	7.3876225	-5.010253	2.78687512	0.00149557	2.44297692891196
HSPA4	-0.643872	6.88249	-5.008298	2.80171960	0.00150140	2.43785651553789
QRICH1	-0.663868	9.16091061	-5.007295	2.80936858	0.00150337	2.4352287312679
CSF3R	0.9539166	9.42833480	5.00647177	2.81565881	0.00150460	2.43307309106019
RNF7	0.62011908	9.55123103	4.99843201	2.87785871	0.00152919	2.41201353790342
PHF15	-0.653808	8.13513307	-4.994251	2.91074499	0.00154232	2.40106255108634
PTTG3	0.64798619	7.12597342	4.99333164	2.91802907	0.00154402	2.39865376139603
FCGR1A	1.16483159	7.756483	4.98520533	2.98319436	0.00156752	2.37736837048097
ABCA1	1.17448991	9.60148984	4.98060596	3.02072030	0.00158209	2.36532144855851
DPY30	0.67246930	8.35647407	4.97967978	3.02833389	0.00158244	2.36289557045058
LOC388344	-1.035529	8.38433473	-4.971483	3.09656002	0.00160910	2.3414259355057
LCN2	2.40545105	9.74658573	4.97099714	3.10064942	0.00160910	2.34015417219884
LOC400759	-0.731979	7.44357826	-4.967164	3.13312583	0.00161791	2.3301135997931
B3GALT4	0.62935348	7.94653611	4.96666394	3.13738339	0.00161791	2.32880505027559
WDR54	-0.757019	8.41828219	-4.966622	3.13773759	0.00161791	2.32869627076414
HS.562660	-0.677653	7.29462973	-4.966469	3.13904494	0.00161791	2.32829486237019
SLC11A1	0.66248136	6.46740280	4.96177536	3.17934850	0.00163645	2.31600159744245
RANGAP1	-0.700336	8.57172892	-4.956719	3.22334907	0.00165684	2.30275775915363
FNBP1	-0.652469	11.4779138	-4.950174	3.28119959	0.00167745	2.28561827542221
SLC35B1	0.66484095	8.55048511	4.94716246	3.30816845	0.00168720	2.27773140656555
RNF135	0.61525697	8.27895861	4.94461540	3.33115065	0.00169579	2.2710611013433
HNRNPD	-0.760675	10.5159763	-4.943693	3.33951575	0.00169579	2.26864467650783
ATP6V1D	0.84299326	9.02821165	4.94088263	3.36512027	0.00170348	2.26128583000141
IER3	1.26102643	7.74351207	4.92852542	3.48006715	0.00175130	2.22892660989236
YIF1B	0.66287065	7.54739342	4.92615588	3.50255329	0.00175966	2.22272187707858
PHIP	-0.667162	9.7713365	-4.925406	3.50969660	0.00176090	2.22075913589596
N4BP2L1	-0.606092	7.14780819	-4.920959	3.55238392	0.00177467	2.20911291424431
CD4	-0.887039	7.80635719	-4.920593	3.55591875	0.00177467	2.20815481805771
PDLIM7	0.78619820	9.07977980	4.91674524	3.59330059	0.00179096	2.19808070155213
ZNF438	0.81478323	7.50545092	4.91614085	3.59920847	0.00179154	2.19649819475889
CDCA5	0.86577427	6.67177630	4.91482030	3.61215060	0.00179561	2.19304055626301
MRPS18C	0.95901233	7.82792438	4.91257861	3.63422701	0.00180163	2.18717111786246
GADD45A	0.89131945	7.19817430	4.91214056	3.63855673	0.00180163	2.18602417325577
LOC124446	0.66445305	9.14652053	4.91052589	3.65456069	0.00180512	2.18179656174152
C7ORF53	0.75854962	6.85116288	4.90788516	3.68088648	0.00181545	2.1748825371129
COX7C	1.30062015	9.78324853	4.90623570	3.69742632	0.00182123	2.17056393663436
HNRNPA0	-0.643731	8.92751207	-4.892677	3.83622983	0.00187010	2.13506707212208
SH3GLB1	1.00396970	10.0471599	4.88966657	3.86774975	0.00187819	2.12718582949412
MCOLN2	-0.809359	7.34592773	-4.888738	3.87752878	0.00188052	2.12475376343035
ANXA3	0.98045384	6.94498038	4.88337017	3.93451113	0.00190373	2.11070329309642
MTF2	-0.633816	8.10405930	-4.883259	3.93569545	0.00190373	2.11041343496237
HLA-DMA	-0.869563	10.2325376	-4.879327	3.97798875	0.00191939	2.10011929137352
AAK1	-0.61291	6.98655107	-4.878439	3.98759754	0.00192157	2.0977958205053
PRNP	-0.780112	9.87521257	-4.87439	4.03172251	0.00194036	2.08719766985026
DAZAP2	-0.593863	13.0910770	-4.869179	4.08923778	0.00196530	2.07355661226921
SLC38A10	0.71653076	7.02356011	4.86875431	4.09395837	0.00196530	2.07244557193428
PNPO	-0.700542	8.75372569	-4.855585	4.24314263	0.00202090	2.03797961304411
NDUFA4	1.08981805	10.1308310	4.85248445	4.27905488	0.00203087	2.02986430467167

MRPL12	0.7039191[illegible]	7.4232572[illegible]	4.8504194[illegible]	4.3031380[illegible]	0.0020372[illegible]	2.02446023847152
TNFRSF1A	0.6384939[illegible]	10.839005[illegible]	4.8404584[illegible]	4.421223[illegible]	0.0020846[illegible]	1.99839426997393
YBX1	-1.304525	11.509166[illegible]	-4.840119	4.4253075[illegible]	0.0020846[illegible]	1.99750529863879
TAF15	-0.849304	10.398659[illegible]	-4.838057	4.4501697[illegible]	0.0020885[illegible]	1.99211125839674
LOC643310	-1.082334	10.252539[illegible]	-4.836722	4.4663457[illegible]	0.0020935[illegible]	1.98861795574008
OAS2	-1.128383	9.8568866[illegible]	-4.834889	4.488658[illegible]	0.0020987[illegible]	1.98382021126584
HSPH1	-0.742618	7.9628713[illegible]	-4.83138	4.531663[illegible]	0.0021136[illegible]	1.97464009292183
PIK4CA	-0.672666	9.3896153[illegible]	-4.828254	4.570328[illegible]	0.0021290[illegible]	1.96646068306772
ANKRD22	1.1774297[illegible]	6.9561962[illegible]	4.8254733[illegible]	4.6049940[illegible]	0.0021425[illegible]	1.95918613420718
DENND2D	-0.690263	9.3727241[illegible]	-4.817546	4.705272[illegible]	0.0021794[illegible]	1.93844793179041
TXNL1	-0.624674	7.3577172[illegible]	-4.817381	4.707392[illegible]	0.0021794[illegible]	1.9380141777182
G6PD	0.6831735[illegible]	8.9097528[illegible]	4.8157099[illegible]	4.7288122[illegible]	0.0021867[illegible]	1.9336439526642
LOC402251	-0.795248	12.017890[illegible]	-4.812116	4.7752177[illegible]	0.0022054[illegible]	1.92424347266639
C3ORF28	-0.671403	8.0416788[illegible]	-4.810244	4.799575[illegible]	0.0022140[illegible]	1.91934594545585
FLJ22662	1.0128476[illegible]	11.887169[illegible]	4.8034822[illegible]	4.8885824[illegible]	0.0022521[illegible]	1.90165870668767
SPRYD3	-2.262263	12.333602[illegible]	-4.799175	4.9461366[illegible]	0.0022677[illegible]	1.89039284976596
VWCE	-1.744233	8.7440354[illegible]	-4.796928	4.9764223[illegible]	0.0022761[illegible]	1.88451729654914
NKG7	-1.142907	12.089221[illegible]	-4.793808	5.0187964[illegible]	0.0022927[illegible]	1.87635643618793
DDX24	-0.729851	8.3299838[illegible]	-4.792589	5.0354454[illegible]	0.0022975[illegible]	1.87316888440021
PID1	-0.678742	6.9717304[illegible]	-4.791587	5.0491763[illegible]	0.0023010[illegible]	1.87054797157092
C1ORF19	-0.645922	7.5809309[illegible]	-4.78684	5.1147263[illegible]	0.0023225[illegible]	1.85813365022139
MPHOSPH10	-0.592295	7.4137796[illegible]	-4.783455	5.1619792[illegible]	0.0023360[illegible]	1.84928314542468
BIN2	-0.58604	7.2923279[illegible]	-4.783385	5.1629729[illegible]	0.0023360[illegible]	1.8490978858709
GZMH	-1.649997	9.6801063[illegible]	-4.782266	5.1786851[illegible]	0.0023375[illegible]	1.84617352297783
IFIT2	-1.332373	10.920244[illegible]	-4.781334	5.1918221[illegible]	0.0023406[illegible]	1.84373528682612
TOP2A	0.7314751[illegible]	6.3791535	4.7790457[illegible]	5.2242005[illegible]	0.0023524[illegible]	1.83775214239912
IL7R	-1.106886	10.796403[illegible]	-4.770201	5.3512575[illegible]	0.0023982[illegible]	1.81462747041902
TOP3B	-0.67385	8.0023946[illegible]	-4.764225	5.4388582[illegible]	0.0024317[illegible]	1.79900240451429
NT5DC2	0.6288411[illegible]	6.5431121[illegible]	4.7633823[illegible]	5.451321[illegible]	0.0024344[illegible]	1.79679983893574
GSTP1	-0.758271	11.225397[illegible]	-4.755036	5.576343[illegible]	0.0024785[illegible]	1.77498138304399
MS4A6A	1.1015774	10.321698[illegible]	4.7506653[illegible]	5.6429458[illegible]	0.0025007[illegible]	1.7635576389536
ORM1	1.5961922[illegible]	7.9984612[illegible]	4.7504528[illegible]	5.646204[illegible]	0.0025007[illegible]	1.76300219488584
HLA-DQB1	-1.144317	7.4017395[illegible]	-4.749821	5.6559104[illegible]	0.0025021[illegible]	1.76134965590086
ZNF689	-0.639833	8.8346623[illegible]	-4.736262	5.8681019[illegible]	0.0025838[illegible]	1.72591568099846
TCN1	1.8993965[illegible]	8.5616951[illegible]	4.7330087[illegible]	5.9201940[illegible]	0.0025976[illegible]	1.71741328483725
RBM25	-0.713205	8.6541675[illegible]	-4.727167	6.0148827[illegible]	0.0026270[illegible]	1.70214878558454
LOC143543	-0.664433	7.4854354[illegible]	-4.723342	6.0776980[illegible]	0.0026473[illegible]	1.69215491210464
ATP5C1	0.8778328[illegible]	8.4876243[illegible]	4.7230676[illegible]	6.0822273[illegible]	0.0026473[illegible]	1.69143830735562
SF3A2	-0.8717	9.9979120[illegible]	-4.722589	6.090140[illegible]	0.0026477[illegible]	1.69018758243193
LOC441246	0.7448365[illegible]	11.316281[illegible]	4.7205249[illegible]	6.124377[illegible]	0.0026564[illegible]	1.68479513891945
C14ORF94	0.7224856[illegible]	7.4373341[illegible]	4.7200410[illegible]	6.1324309[illegible]	0.0026569[illegible]	1.68353105986874
ATG9A	0.7255058[illegible]	8.0575776[illegible]	4.7178657[illegible]	6.1687691[illegible]	0.0026696[illegible]	1.67784804783765
SYF2	1.0520755	7.6568445[illegible]	4.7172337[illegible]	6.1793676[illegible]	0.0026711[illegible]	1.67619684524076
C20ORF3	0.7784935[illegible]	9.6379025[illegible]	4.7118437[illegible]	6.2704887[illegible]	0.0027074[illegible]	1.66211670884023
SERPINA1	0.6352774[illegible]	6.6981537[illegible]	4.7081651[illegible]	6.3334466[illegible]	0.0027264[illegible]	1.65250776515413
BBS2	-0.597942	7.5598093[illegible]	-4.698945	6.4940353[illegible]	0.0027790[illegible]	1.62842531104098
RPL27	0.9167353[illegible]	11.874939[illegible]	4.6988705[illegible]	6.4953427[illegible]	0.0027790[illegible]	1.62823171000691
RBM14	-0.663486	9.5112538[illegible]	-4.698232	6.5066088[illegible]	0.0027800[illegible]	1.62656503655859
OLAH	1.6722897[illegible]	6.8564189[illegible]	4.6960259[illegible]	6.5457103[illegible]	0.0027880[illegible]	1.62080284127611
GALNT4	0.6177594[illegible]	7.0467277[illegible]	4.6955322	6.5544914[illegible]	0.0027886[illegible]	1.61951356036093
PLOD2	0.6303545[illegible]	6.5554358[illegible]	4.6866245[illegible]	6.7149598[illegible]	0.0028519[illegible]	1.59625323610898

HLA-G	-1.430121	9.8599595	-4.686438	6.7183626	0.0028519	1.5957660540104
LOC650518	0.9003341	9.1003233	4.6844947	6.7539045	0.0028617	1.59069220776236
KIFC1	0.6998554	6.5882776	4.6843180	6.7571464	0.0028617	1.59023073888395
TYMS	1.1741070	7.0755467	4.6835174	6.7718498	0.0028617	1.58814055224126
BPI	2.1324262	7.4390540	4.6821749	6.7965785	0.0028689	1.58463546001179
TOR3A	0.6503207	7.7800368	4.6810294	6.8177507	0.0028730	1.5816446229122
SCRN1	-0.602756	7.6528936	-4.680536	6.8268948	0.0028730	1.5803557893635
VAMP5	0.5914549	9.3651586	4.6795466	6.8452530	0.0028766	1.57777346995304
RGL4	1.1713747	9.3634502	4.6744782	6.9400991	0.0029100	1.56454183618067
BRSK1	0.6618183	8.3178950	4.6726159	6.9752774	0.0029139	1.55968030939981
PAF1	-0.61862	8.6078763	-4.671534	6.9957887	0.0029172	1.55685705658665
SNRPG	1.0926117	9.2510203	4.6703561	7.0182018	0.0029221	1.55378150805501
LOC645317	0.6983606	8.1770670	4.6701003	7.0230783	0.0029221	1.55311366155387
LOC647340	1.0220895	9.3494978	4.6679479	7.0642350	0.0029360	1.54749557764461
POMP	0.6340353	10.560300	4.6633725	7.1525268	0.0029629	1.53555331429946
SEMA4A	0.7372960	9.0318469	4.6595262	7.2275966	0.0029875	1.52551519800094
C6ORF125	0.7910149	7.0805315	4.6580804	7.2560186	0.0029959	1.52174197628594
DOCK2	-0.59638	11.101297	-4.657444	7.2685554	0.0029978	1.5200823319867
FCER1A	-1.108848	8.1824231	-4.646555	7.4866062	0.0030677	1.49166693802266
FBN2	0.6748203	7.1049394	4.6441981	7.5346454	0.0030786	1.48551837461888
C16ORF68	0.8280016	8.0248575	4.6439701	7.5393080	0.0030786	1.48492369430635
NGFRAP1	0.8145021	6.9834428	4.6436544	7.5457723	0.0030786	1.4840998427111
MRPS24	0.5932218	8.8253827	4.6408936	7.6025227	0.0030950	1.47689744502164
EBI2	-0.772288	8.6457030	-4.639912	7.6228094	0.0030979	1.47433586927593
GMFG	0.7088513	11.166676	4.6397623	7.6259014	0.0030979	1.47394604832676
CST7	1.3332278	10.877814	4.6384375	7.6533690	0.0031057	1.47049002157276
IPO11	0.8721105	7.5572107	4.6357174	7.7100746	0.0031253	1.46339443140899
SAR1B	0.7535568	7.6572586	4.6306156	7.8175634	0.0031554	1.45008686761966
C20ORF24	0.6440299	12.540705	4.6189592	8.0687800	0.0032360	1.41968777377126
GPR175	-2.248777	11.706904	-4.609462	8.2793819	0.0033170	1.39492640678252
UBB	-1.348151	13.547043	-4.608571	8.2994337	0.0033201	1.39260187897018
PFKFB2	1.0087541	6.5618256	4.5989933	8.5178943	0.0033945	1.36763554764577
LOC440567	0.9747259	10.795833	4.5947831	8.6157282	0.0034222	1.35666241349702
LTA	-0.621403	7.7604072	-4.59274	8.6636146	0.0034309	1.35133694415752
ELL2	0.6012596	6.7070483	4.5885990	8.7614625	0.0034624	1.3405464577132
METTL9	0.8260333	7.6232566	4.5843367	8.8633347	0.0034953	1.3294399298972
LOC649841	-0.853989	7.8493623	-4.583083	8.8935329	0.0035036	1.32617220754403
LOC440313	-2.608429	11.240746	-4.572414	9.1545859	0.0035952	1.29837944272993
CSDA	-1.187483	13.620384	-4.568772	9.2454542	0.0036271	1.28889151097918
GVIN1	-1.32184	10.549094	-4.562746	9.3977622	0.0036792	1.27319638285225
LMNB1	0.8798737	7.7794902	4.5619706	9.4175303	0.0036793	1.27117804577713
ALS2CR13	-0.72843	9.1362061	-4.558882	9.4967295	0.0037026	1.26313419678569
PCBP2	-0.624063	12.287112	-4.557873	9.5227274	0.0037089	1.26050839668393
TTC3	-0.684057	9.0913329	-4.555037	9.5962334	0.0037333	1.25312300367939
LOC648984	1.2449937	7.8969387	4.5525793	9.6603818	0.0037471	1.24672405019521
CD151	0.6733938	6.7961721	4.5516254	9.6853949	0.0037491	1.24424048449617
ZNF559	-0.635448	7.1064769	-4.550472	9.7157200	0.0037570	1.24123810948183
CLEC4D	0.7886669	6.6519533	4.5497700	9.7342322	0.0037581	1.23940989670043
WASPIP	-0.838251	10.534305	-4.549603	9.7386470	0.0037581	1.23897442658429
HPS6	-0.621117	9.3789240	-4.544876	9.8642334	0.0037988	1.22666887163816
SSBP2	-0.633808	8.1981271	-4.538973	0.0001002	0.0038483	1.21130571084911

ATP5O	0.9427624	9.6588248	4.5367731	0.0001008	0.0038674	1.20557898810974
FAM113B	-0.96987	9.6676122	-4.534967	0.0001013	0.0038825	1.2008775332099
GPBAR1	-0.741551	8.1566595	-4.527932	0.0001032	0.0039452	1.18257271142951
MTF1	0.9001637	9.1070347	4.5202823	0.0001054	0.0040076	1.16266978860284
C14ORF159	-0.658086	9.3654643	-4.511338	0.0001080	0.0041017	1.13940376022503
BIN1	-0.966041	9.6589596	-4.504325	0.0001100	0.0041720	1.12116660472675
CAPZA2	0.9477772	10.325776	4.4949970	0.0001129	0.0042604	1.09691447701995
AMY1B	-0.693089	7.9925225	-4.49474	0.0001129	0.0042604	1.09624653508511
SH2B2	0.5891334	6.7432860	4.4898182	0.0001145	0.0043132	1.0834525299939
GINS2	0.7882472	6.6805577	4.4890475	0.0001147	0.0043179	1.08144929704864
PSMA6	0.8829797	8.3744264	4.4694033	0.0001210	0.0045223	1.03040487672982
ID3	-0.722365	7.5094338	-4.466771	0.0001218	0.0045456	1.02356731179491
RHOB	-0.697996	8.5557214	-4.465292	0.0001223	0.0045584	1.01972498838332
SF3B14	0.8143932	8.4794306	4.4605776	0.0001239	0.0045998	1.00748134962659
HS.407903	1.1626295	9.3005158	4.4568284	0.0001252	0.0046331	0.997745113528522
MOSPD2	0.6257421	9.1395312	4.4560132	0.0001254	0.0046344	0.995628170163008
ELA2	2.5496219	8.1228993	4.4534040	0.0001263	0.0046496	0.988853321508844
ANPEP	0.7667929	9.4078834	4.4508914	0.0001272	0.0046677	0.982329814677032
CEBPD	0.7157237	11.989403	4.4469165	0.0001286	0.0047037	0.972010491903109
FXYD5	-0.648287	10.390853	-4.444976	0.0001292	0.0047207	0.966973910599918
SAMSN1	1.1970890	7.5414890	4.4437793	0.0001297	0.0047301	0.963866895196358
DEFA4	2.623983	8.4595928	4.4396878	0.0001311	0.0047689	0.953247566119092
HNRNPUL2	-0.653044	9.3161148	-4.43792	0.0001317	0.0047826	0.948659423163702
CDC20	0.9725695	6.8260662	4.4370525	0.0001320	0.0047892	0.946408165005352
AIM2	1.0413012	7.8102736	4.4317427	0.0001340	0.0048399	0.932629928535526
C16ORF61	0.6288202	8.2805545	4.4286617	0.0001351	0.0048758	0.924636031651692
HSPC159	0.6216324	6.8432555	4.4263732	0.0001359	0.0049014	0.918698892186453
CNOT8	-0.597626	7.6877733	-4.42586	0.0001361	0.0049035	0.917366961630679
BHLHB2	-0.655569	9.9227158	-4.424528	0.0001366	0.0049165	0.913911447968974
LOC644039	-0.616089	13.722744	-4.422265	0.0001374	0.0049420	0.908041271380218
GGH	0.9081413	6.9080798	4.4213901	0.0001378	0.0049490	0.905772482844187
HS.554324	-0.91999	9.9470826	-4.417387	0.0001393	0.0049886	0.89539020952079
SLC20A1	-0.594615	9.7709394	-4.411588	0.0001415	0.0050579	0.880351428380039
ALPL	1.1876958	12.159270	4.4030144	0.0001448	0.0051570	0.858123636044255
BATF	0.7974260	7.5675416	4.4004452	0.0001458	0.0051783	0.851463805709805
SERPINA13	-2.710821	10.901317	-4.39591	0.0001476	0.0052323	0.839709413728809
LOC652071	-0.585405	13.704187	-4.395146	0.0001479	0.0052382	0.837729579335837
OGT	-0.617158	10.582443	-4.393305	0.0001486	0.0052427	0.832959415119105
RABGEF1	0.6715731	6.7641291	4.3931061	0.0001487	0.0052427	0.832443105575393
LOC728888	-0.655868	10.517715	-4.392898	0.0001488	0.0052427	0.831903158730783
GBP5	-0.897181	8.9939652	-4.390065	0.0001499	0.0052712	0.824562575036918
TMEM120A	1.0724597	7.9135734	4.3881263	0.0001507	0.0052939	0.819539617514538
TNFRSF10B	-0.600588	8.8789837	-4.386104	0.0001516	0.0053180	0.814300491043463
FAM62A	-0.966926	9.8509244	-4.381839	0.0001533	0.0053498	0.80325209687999
TKT	0.7369852	12.466799	4.3763109	0.0001556	0.0054104	0.788932998251211
MS4A7	-0.940111	8.9692965	-4.375456	0.0001560	0.0054179	0.786718084837807
DKFZP761P	-0.606159	7.6064618	-4.363107	0.0001613	0.0055710	0.754745453830547
FURIN	0.7038431	7.9231280	4.3560985	0.0001644	0.0056438	0.736604415833941
CCDC109B	-0.590104	9.9654861	-4.355937	0.0001644	0.0056438	0.736185521394963
OPLAH	1.0764017	7.1672929	4.3474580	0.0001682	0.0057588	0.714246500505583
DNAJC3	0.6028153	6.3962123	4.3457150	0.0001690	0.0057807	0.70973719776497

DDAH2	0.61381272	6.85737373	4.34531547	0.00016926	0.00578178	0.708703481887342
MRPL51	0.76684891	8.78804834	4.34373609	0.00016998	0.00579600	0.704617805308604
RPL15	-0.669183	10.5708082	-4.342765	0.00017042	0.00579814	0.702106552279038
CMPK1	-0.686936	8.59409607	-4.342598	0.00017050	0.00579814	0.701674085841917
FAM100B	0.65716504	8.93983296	4.34007397	0.00017167	0.00582729	0.695145257428195
ENY2	0.95230541	8.08530288	4.32451681	0.00017903	0.00604459	0.654919223369775
CEACAM1	0.80339083	7.58873634	4.32293637	0.00017979	0.00605960	0.650834038825545
GIMAP4	-0.698743	11.9592543	-4.315207	0.00018358	0.00617077	0.630859602647273
TARP	-0.672746	7.03171596	-4.309956	0.00018620	0.00624770	0.61729090476167
SLC9A8	0.73514877	7.30571984	4.30739015	0.00018750	0.00627993	0.610662799106855
LOC651143	0.63968990	7.39287388	4.30574157	0.00018833	0.00629675	0.606404340553408
C11ORF82	0.62044968	6.74017753	4.30318726	0.00018963	0.00632543	0.599806817413608
CDC45L	0.77289379	6.46060180	4.30249307	0.00018999	0.00632976	0.598013909243628
HS.452445	-0.777654	8.21950265	-4.297587	0.00019252	0.00640276	0.585345193616851
UQCRC1	0.58795601	8.63085165	4.28600971	0.00019862	0.00655385	0.555456507325751
C19ORF10	0.78836494	7.19747088	4.28043516	0.00020163	0.00662496	0.541070398998611
MAP2K1IP1	0.72444877	8.44169253	4.27983078	0.00020196	0.00662918	0.539510875566415
QPCT	0.89520874	11.0417168	4.27767532	0.00020314	0.00665623	0.533949337739287
NDUFS5	0.93525761	8.85338946	4.27487238	0.00020468	0.00669580	0.526717932177643
SQSTM1	-0.642862	10.1213176	-4.270634	0.00020703	0.00675445	0.515784287411346
IGFBP7	0.61192843	7.11623461	4.26770371	0.00020867	0.00679040	0.508227040266905
LOC731365	0.66067790	11.6549532	4.26280932	0.00021144	0.00684612	0.495605652087118
SULT1B1	0.7634323	7.07257323	4.26275696	0.00021147	0.00684612	0.495470638114999
MXD3	0.71856023	7.04162834	4.26163234	0.00021211	0.00686102	0.492570905915904
CDKN1C	-0.729912	7.13903111	-4.259462	0.00021335	0.00688703	0.48697642028256
SFMBT2	-0.644642	7.06834607	-4.251904	0.00021774	0.00698931	0.467493128092331
OSM	0.70079023	7.07386265	4.24827996	0.00021988	0.00702800	0.458153637557704
SORT1	0.78010238	7.87990811	4.24414999	0.00022233	0.00708262	0.44751216632708
GCN1L1	-0.594968	7.89092273	-4.240549	0.00022450	0.00713959	0.438234428070825
CMTM5	1.14030129	7.51879926	4.23215229	0.00022963	0.00727219	0.416609351643256
VSTM1	1.14322844	7.29571065	4.22808188	0.00023216	0.00732772	0.406128807630791
LOC389599	-2.051049	11.3770286	-4.227648	0.00023243	0.00733017	0.405010474345037
KLRG1	-0.939746	8.62835219	-4.212219	0.00024229	0.00759647	0.365302356883262
AHCY	0.60091437	8.50218373	4.21122414	0.00024293	0.00761050	0.362743729388633
DPYSL2	-0.816865	10.3593653	-4.205581	0.00024665	0.00769499	0.348227858927897
TMEM119	1.12464681	6.55939773	4.19652374	0.00025273	0.00785227	0.324938109838786
LTBR	0.6983029	8.57361542	4.19454893	0.00025408	0.00786313	0.319861393641012
MUC6	-2.156377	10.9928433	-4.188708	0.00025810	0.00793417	0.304848506450394
KIAA1881	0.65801788	6.82035115	4.18210161	0.00026272	0.00806317	0.287873446721728
IL32	-0.755461	8.16851861	-4.177164	0.00026623	0.00813122	0.275190964552846
MAGMAS	0.63761281	8.19768611	4.17375438	0.00026868	0.00819814	0.266432839180457
LBH	-0.910176	7.68292907	-4.173512	0.00026885	0.00819814	0.265810941684976
SGSM2	-0.6595	9.08040673	-4.170211	0.00027125	0.00825784	0.257333365152372
IL10RB	0.73626311	9.88518838	4.16688398	0.00027368	0.00829873	0.248792121052215
D4S234E	-0.742661	7.32850307	-4.16688	0.00027369	0.00829873	0.248782743928693
RPL5	-0.612833	11.4539502	-4.161376	0.00027776	0.00840781	0.234655072624457
TXNDC5	0.62462845	6.36898153	4.16111706	0.00027796	0.00840781	0.233989284588375
SBNO2	0.72720574	8.17334061	4.16019517	0.00027864	0.00842190	0.231623325282954
TAF12	0.62106058	7.60523673	4.15972177	0.00027900	0.00842588	0.23040841252465
CHST7	-0.660662	9.10177792	-4.158822	0.00027967	0.00842600	0.228100468914403
FKBP5	1.25014569	9.37676126	4.15821810	0.00028013	0.00842600	0.226549667707225

FAM14A	0.67433320	7.71965480	4.15762853	0.00028057	0.00842622	0.225036801420452
HNRPM	-0.703156	10.6088535	-4.142432	0.00029225	0.00870097	0.186056250359425
CLEC16A	-0.661618	8.18337488	-4.140217	0.00029400	0.00873906	0.180377831895819
ETS2	0.81683143	8.47540569	4.13684384	0.00029667	0.00881159	0.171730070483012
ATP5L	0.79428731	10.7231991	4.12464218	0.00030654	0.00902039	0.140462566398833
MMRN1	0.66345719	6.77786673	4.11567175	0.00031400	0.00916078	0.117487875290393
FCGR1B	1.27178720	8.08591380	4.11417484	0.00031527	0.00918344	0.113655099546918
HAGH	-1.48754	11.4693541	-4.108822	0.00031982	0.00928038	0.099951950322672
CD3G	-0.904447	7.55054192	-4.0976	0.00032959	0.00946196	0.0712360942503336
PHACTR4	-0.717853	8.33870669	-4.091473	0.00033504	0.00956766	0.0555651761475131
Septin 6	-0.652073	8.41067423	-4.088976	0.00033729	0.00961732	0.0491801066095263
FTHL12	-0.620237	12.5943745	-4.087988	0.00033818	0.00963554	0.0466531158098515
RAB31	0.81742156	11.4064223	4.08715378	0.00033894	0.00964254	0.0445213188521913
LOC646766	0.70767173	10.5525615	4.07396126	0.00035113	0.00985450	0.0108048444201936
CA4	1.26477955	9.57101738	4.07372706	0.00035135	0.00985450	0.0102065081149298
ITGAM	0.66703223	10.0754485	4.06892457	0.00035589	0.00995243	-0.002061
RPL14	-1.064037	8.97482603	-4.06342	0.00036117	0.01007774	-0.016118
TSHZ3	0.68045002	7.85497446	4.06074944	0.00036376	0.01014251	-0.022936
LIME1	-0.741217	10.597601	-4.06026	0.00036424	0.01014832	-0.024187
SIGLEC9	0.59432944	6.74288165	4.05781576	0.00036663	0.01019233	-0.030425
CARM1	-1.163529	10.8674522	-4.051613	0.00037276	0.01031728	-0.046254
PQLC3	-0.598826	8.97541211	-4.050262	0.00037411	0.01034098	-0.049701
CHMP5	0.75143868	7.31614134	4.04463213	0.00037979	0.01045806	-0.064063
PTPLA	0.59570131	6.68341526	4.04353428	0.00038090	0.01045844	-0.066863
DR1	-0.649093	7.84432626	-4.042471	0.00038199	0.01048048	-0.069576
CSF2RA	0.71416698	8.47443357	4.04149094	0.00038299	0.01049693	-0.072074
ASGR2	0.68894094	7.27639488	4.04002332	0.00038449	0.01052017	-0.075817
FAM116B	-1.622775	10.0651704	-4.031454	0.00039340	0.01072358	-0.097663
AMPH	0.83025305	6.58485892	4.03001452	0.00039492	0.01073792	-0.101331
RAB24	0.68446109	8.92541469	4.02981113	0.00039513	0.01073792	-0.101849
PARK7	0.95484661	8.22554334	4.02960621	0.00039535	0.01073792	-0.102371
DPYSL5	-2.204214	10.5813431	-4.026847	0.00039827	0.01078635	-0.109402
RPS17	0.88103572	12.0207851	4.02531009	0.00039991	0.01080749	-0.113318
HPSE	0.94293287	8.19106619	4.01908075	0.00040662	0.01098098	-0.129185
GZMM	-0.755778	8.00681184	-4.017532	0.00040831	0.01101861	-0.133129
PTPLAD2	-0.982791	10.8967995	-4.012431	0.00041391	0.01115071	-0.146117
F5	0.84743013	7.58994784	4.01226765	0.00041409	0.01115071	-0.146533
NSUN7	0.75104499	6.97087176	4.00653693	0.00042047	0.01128241	-0.161119
LOC651436	1.08498404	9.95009757	4.00385777	0.00042349	0.01133922	-0.167937
KDELR2	0.62899905	8.59351911	4.00141952	0.00042626	0.01138904	-0.17414
TFF3	1.16918651	7.17356230	3.99469678	0.00043397	0.01153807	-0.191239
METTL7A	-0.610031	8.82463157	-3.99329	0.00043560	0.01157332	-0.194817
LOC644250	-0.968837	10.9538491	-3.990072	0.00043936	0.01163221	-0.203
MYBPC3	0.83959188	7.37626257	3.98285278	0.00044790	0.01182498	-0.221348
CCR6	-0.652038	7.78228646	-3.976389	0.00045569	0.01193957	-0.23777
ATP8B4	1.00160304	8.03643488	3.96584921	0.00046867	0.01221978	-0.264533
MRPL53	0.64871579	7.82818969	3.96107506	0.00047467	0.01229138	-0.27665
TMCC2	1.03646448	7.23074403	3.95838241	0.00047808	0.01233653	-0.283482
DGCR2	0.67819249	7.80773561	3.95555340	0.00048170	0.01239338	-0.290659
LOC644863	0.69705569	9.60370773	3.94686473	0.00049298	0.01262698	-0.312694
CDC123	0.63342502	8.70394823	3.94520650	0.00049516	0.01266567	-0.316898

TPT1	0.84482565	11.8617250	3.94388799	0.00049690	0.01270160	-0.32024
SULT1A1	0.65337434	8.12556303	3.94118662	0.00050048	0.01278461	-0.327087
PCOLCE2	0.68354431	6.44066738	3.93059947	0.00051478	0.01306157	-0.353909
ZNF800	-0.598301	7.64354153	-3.924966	0.00052256	0.01319674	-0.368175
IL4R	0.70101070	8.57668526	3.92382652	0.00052414	0.01321910	-0.371059
CCR7	-1.002205	9.67233007	-3.920129	0.00052932	0.01332303	-0.380417
HS.444999	-0.62577	7.49806242	-3.917254	0.00053338	0.01339848	-0.387693
HS.143018	-0.844016	9.77540934	-3.911974	0.00054092	0.01356082	-0.401051
ZNF608	0.65311936	6.45122419	3.90766264	0.00054715	0.01368983	-0.411954
C16ORF7	0.77212811	7.99567926	3.90312327	0.00055379	0.01378292	-0.423431
KCNH3	0.73634402	7.11118211	3.89736145	0.00056233	0.01392549	-0.437994
NUSAP1	0.72960013	7.03074496	3.89150272	0.00057115	0.01411267	-0.452795
LOC731314	0.64700665	7.63640119	3.88976900	0.00057379	0.01414662	-0.457174
ATP5G1	0.60476456	9.32740696	3.88972496	0.00057385	0.01414662	-0.457285
GPR84	0.82378158	6.78179742	3.8877	0.00057695	0.01420015	-0.462399
MGEA5	-0.704603	11.1036087	-3.886877	0.00057821	0.01422196	-0.464478
CROP	-0.601063	7.30030876	-3.877163	0.00059331	0.01454601	-0.488998
GRN	0.83705527	10.2705606	3.87530387	0.00059624	0.01459898	-0.493688
SLC44A1	0.67705586	8.46652876	3.87455506	0.00059745	0.01460907	-0.495577
MYO1F	0.79855530	9.07822326	3.86994406	0.00060478	0.01476973	-0.507208
ZNF217	-0.591508	9.61510680	-3.863195	0.00061570	0.01495908	-0.524225
NACA	-0.591313	12.3396081	-3.857632	0.00062485	0.01515209	-0.538246
MS4A3	1.57380466	7.61115092	3.84745571	0.00064193	0.01546697	-0.563879
ZNF223	-0.903731	10.2333173	-3.845588	0.00064511	0.01552390	-0.568582
CCL4L1	-0.738841	7.4111835	-3.843574	0.00064856	0.01557714	-0.573651
POLD4	0.61070666	7.61760307	3.84168009	0.00065182	0.01562568	-0.578419
CASP5	0.83419461	6.92899715	3.83846347	0.00065740	0.01572941	-0.586514
CDA	0.59925874	10.2559773	3.83783598	0.00065849	0.01574560	-0.588093
STAB1	0.68140151	6.71805711	3.83737015	0.00065931	0.01575506	-0.589265
C16ORF57	0.58644993	8.31143557	3.82938992	0.00067338	0.01603059	-0.609338
EXOC6	0.68432658	7.49681719	3.82275676	0.00068531	0.01625299	-0.626013
RBX1	1.01023019	9.59781161	3.81818176	0.00069365	0.01638916	-0.63751
BANK1	-0.628898	7.92117965	-3.817707	0.00069452	0.01639949	-0.638702
TSPAN9	0.84861009	8.66054857	3.81647721	0.00069678	0.01644265	-0.641792
C21ORF55	-0.636228	13.8735484	-3.810576	0.00070774	0.01665961	-0.656613
TGFBI	-1.048815	10.5094332	-3.808465	0.00071170	0.01673198	-0.661913
FN3KRP	-0.589	8.16019815	-3.799195	0.00072936	0.01701992	-0.68518
ASPSCR1	-0.59911	8.79932819	-3.798381	0.00073092	0.01704496	-0.68722
CTSG	1.93183756	7.24363311	3.78171025	0.00076382	0.01771482	-0.729016
HLA-DRA	-0.872801	11.7931690	-3.779035	0.00076924	0.01778154	-0.735717
HLA-DRB4	-1.128304	10.203226	-3.777586	0.00077218	0.01781031	-0.739348
PDIA3P	-0.754462	7.45989669	-3.771509	0.00078467	0.01804311	-0.754564
IL18	-0.594659	13.5616243	-3.770089	0.00078761	0.01809979	-0.758118
TRPA1	-0.680397	6.71672203	-3.755966	0.00081749	0.01860547	-0.793451
KLRB1	-0.970264	9.72336673	-3.75491	0.00081977	0.01862282	-0.79609
GTPBP4	-0.641238	8.23672007	-3.745661	0.00083999	0.01890655	-0.819204
RUNDC2C	-0.656609	7.57217115	-3.72892	0.00087784	0.01957801	-0.860998
LDHA	0.93208205	11.1388549	3.72583537	0.00088500	0.01970764	-0.868691
APH1B	0.61603286	8.05219846	3.72229656	0.00089328	0.01983672	-0.877516
AGPAT9	0.65780531	9.12798019	3.71885675	0.00090139	0.01996179	-0.886091
TNFSF14	0.71657781	9.54792603	3.71826401	0.00090280	0.01998122	-0.887569

PECR	0.86328393	7.32138326	3.71741600	0.00090481	0.02001410	-0.889682
LAG3	-0.616273	7.00219169	-3.715504	0.00090937	0.02006796	-0.894447
STK11	0.58744290	6.92603038	3.70313904	0.00093941	0.02058177	-0.925244
CCDC72	1.03813951	8.60080965	3.69980402	0.00094768	0.02068544	-0.933544
C5ORF30	0.61029320	6.70172319	3.69941661	0.00094865	0.02068544	-0.934508
DYSF	0.73637798	10.3168297	3.69937184	0.00094876	0.02068544	-0.93462
ATOX1	0.63386226	8.84042865	3.69761159	0.00095315	0.02074327	-0.939
CCPG1	0.76218706	8.86931115	3.69163043	0.00096824	0.02102318	-0.953878
PARP1	-0.608841	9.62877273	-3.687264	0.00097940	0.02125334	-0.964735
ATIC	-0.612812	8.77428276	-3.68649	0.00098140	0.02126426	-0.966659
DAAM2	0.83890790	6.58112473	3.68541416	0.00098417	0.02128340	-0.969332
ATP6V1E1	0.61053744	9.88729723	3.66692739	0.00103306	0.02208791	-1.015243
LGALS3	-1.183877	9.99821880	-3.66457	0.00103947	0.02219170	-1.021091
HLA-DPB1	-0.636885	6.95417161	-3.662951	0.00104389	0.02224376	-1.025108
PLAC8	0.78280372	10.4575479	3.66096037	0.00104935	0.02232736	-1.030046
TNFSF13B	0.69160691	10.5660574	3.65707283	0.00106009	0.02248753	-1.039686
SIL1	0.64831201	7.81929423	3.65466515	0.00106679	0.02260441	-1.045654
P2RX1	0.64413463	7.59639288	3.65127187	0.00107632	0.02276187	-1.054064
HS.82028	-0.671598	8.32781330	-3.650485	0.00107854	0.02278929	-1.056013
MBOAT7	0.68796172	9.90224730	3.63912153	0.00111110	0.02332235	-1.084155
RPL39	0.80341577	11.9121580	3.63799884	0.00111437	0.02337640	-1.086934
SIRPB1	0.66095265	8.30487626	3.63660684	0.00111843	0.02343566	-1.090379
ANXA2P1	-0.720584	8.19426203	-3.63509	0.00112288	0.02347673	-1.094131
PLEK	-0.633999	11.7861051	-3.634713	0.00112398	0.02348689	-1.095064
DDX5	-0.656719	11.628409	-3.631227	0.00113428	0.02363662	-1.103688
GBP1	-0.895444	8.51152357	-3.630284	0.00113708	0.02366890	-1.106021
FLJ42957	0.80606174	7.609914	3.62838296	0.00114275	0.02372149	-1.110722
LSM1	0.70580094	9.41862157	3.62762693	0.00114501	0.02375537	-1.112592
SLC38A1	-0.658831	9.85752207	-3.62325	0.00115818	0.02396291	-1.123411
FCRLA	-0.765257	8.068579	-3.616424	0.00117902	0.02424808	-1.140277
PARP10	0.58668751	7.05473684	3.60179289	0.00122494	0.02490762	-1.176391
LOC441775	0.63131936	9.9082565	3.59711334	0.00123999	0.02506535	-1.187931
AZU1	1.62485326	6.90313396	3.59512061	0.00124645	0.02518239	-1.192844
SLC26A6	0.75944727	6.87377080	3.58928153	0.00126558	0.02547345	-1.207233
C22ORF34	-0.744896	7.48363292	-3.574755	0.00131441	0.02623271	-1.242997
IL18R1	1.30267841	8.30284596	3.57368427	0.00131808	0.02627818	-1.24563
AOAH	0.67803231	8.54918588	3.55603186	0.00138005	0.02728340	-1.289016
PDPR	-0.628417	8.55338396	-3.544235	0.00142304	0.02784762	-1.317968
CACNG6	-0.635586	6.9577625	-3.538622	0.00144395	0.02812029	-1.331731
PRKAR1A	-0.653826	11.3246503	-3.536379	0.00145238	0.02822593	-1.337227
HS.211743	0.81215677	7.06729561	3.53043308	0.00147498	0.02857697	-1.351796
FLJ11795	0.98697048	7.22788911	3.52941383	0.00147889	0.02862580	-1.354292
RPA1	-0.640669	7.86449992	-3.520053	0.00151526	0.02917637	-1.377207
TNFAIP8L1	-0.624761	7.62749807	-3.51645	0.00152949	0.02937685	-1.386021
AQP10	0.919432	7.68238915	3.51460787	0.00153681	0.02947256	-1.390525
SNX10	-0.69384	9.37882146	-3.512679	0.00154452	0.02955849	-1.395241
C22ORF13	-0.879615	11.6184316	-3.512662	0.00154458	0.02955849	-1.395282
LOC401115	0.62052563	10.5228415	3.50905713	0.00155909	0.02974531	-1.404094
C2ORF25	0.61905456	9.77813238	3.50813608	0.00156282	0.02977475	-1.406345
LOC613037	-0.591081	8.93018907	-3.505136	0.00157501	0.02996185	-1.413676
CLEC4A	0.61849779	8.61951984	3.50345349	0.00158190	0.03006246	-1.417785

LOC441193	0.59230509	6.9391355	3.50005838	0.00159587	0.03028235	-1.426076
CD74	-0.863614	11.5513207	-3.499392	0.00159863	0.03031940	-1.427702
NCR3	-0.620699	7.5791722	-3.491117	0.00163325	0.03083680	-1.447898
ANXA1	0.98442143	10.9460503	3.48992944	0.00163828	0.03090108	-1.450793
CDK5RAP2	0.99899969	8.14192157	3.48450082	0.00166146	0.03118229	-1.46403
PHTF1	0.64316084	6.90417492	3.48279305	0.00166881	0.03130481	-1.468192
LRRC33	-0.644503	8.75668303	-3.479278	0.00168405	0.03154374	-1.476758
LOC728519	0.94440669	7.65067473	3.47585910	0.00169901	0.03177656	-1.485085
PFKFB3	0.59858312	7.85942642	3.46827038	0.00173266	0.03218873	-1.503558
BMP2K	-0.649314	7.60606884	-3.466441	0.00174087	0.03223731	-1.508009
LOC400304	-0.692459	7.37506015	-3.464577	0.00174927	0.03233249	-1.512542
CITED4	0.61426495	7.15594226	3.45824304	0.00177812	0.03276954	-1.527944
VAMP2	-0.74175	9.18809769	-3.453308	0.00180092	0.03301254	-1.539937
MAOA	1.16838769	6.67716546	3.45264257	0.00180401	0.03305322	-1.541553
PRTN3	1.31573865	6.83439738	3.44527576	0.00183863	0.03354101	-1.559441
CD177	0.96005334	6.63168330	3.44127224	0.00185770	0.03370544	-1.569156
ANKRD30B	-0.737261	12.5544103	-3.440454	0.00186163	0.03370544	-1.571141
CDC42EP3	0.66898598	6.93429573	3.43879012	0.00186963	0.03381273	-1.575177
CYP27A1	0.85971676	8.72563084	3.43499551	0.00188800	0.03406351	-1.584378
FOXC1	0.66929670	6.49179161	3.43267895	0.00189931	0.03416953	-1.589994
NDUFS4	0.60756915	7.77269007	3.42791774	0.00192274	0.03434585	-1.601531
UHRF1	0.59913138	6.73978623	3.42505491	0.00193697	0.03453225	-1.608465
FTHL2	-0.733696	11.3292147	-3.420868	0.00195796	0.03474499	-1.618603
IL1RAP	-0.659203	7.98217419	-3.420337	0.00196064	0.03475978	-1.619886
ABCA13	0.80741195	6.40552461	3.41919877	0.00196639	0.03479647	-1.622642
ANKRD55	0.92564991	7.18463019	3.41840055	0.00197044	0.03483539	-1.624573
HS.544831	0.58723144	6.47890815	3.40055293	0.00206297	0.03603297	-1.667718
NME2	-0.721257	7.49701534	-3.395611	0.00208933	0.03635884	-1.679648
MX1	-1.180767	11.1411311	-3.393613	0.00210008	0.03652906	-1.684469
SLC2A3	0.63929109	12.7683287	3.39309409	0.00210288	0.03656096	-1.685722
NOL10	-1.617703	9.12377692	-3.388304	0.00212890	0.03684382	-1.697277
MPO	1.65805433	7.14580423	3.38008475	0.00217427	0.03744451	-1.717087
UBE2O	-0.962711	8.45812834	-3.375597	0.00219944	0.03777046	-1.727895
LOC728358	2.08116094	10.8851967	3.37278554	0.00221535	0.03795744	-1.734665
HPGD	0.97830780	6.71770823	3.37086962	0.00222626	0.03807523	-1.739276
PAX7	0.68476880	7.28260803	3.36231476	0.00227559	0.03862171	-1.759852
BCL6	0.89564451	11.5532405	3.35562263	0.00231491	0.03907847	-1.775934
HLA-DRB3	-1.144019	8.55825315	-3.352007	0.00233643	0.03942413	-1.784618
FES	0.69559659	8.9226295	3.35055792	0.00234511	0.03953412	-1.788096
TBC1D8	0.61149444	7.11788219	3.34894015	0.00235484	0.03964610	-1.79198
SIPA1L2	0.75333290	8.28029596	3.34148139	0.00240018	0.04023024	-1.809874
LOC439949	-0.802106	8.85274788	-3.333606	0.00244897	0.04084897	-1.82875
ARL4A	-0.910485	7.30122296	-3.331733	0.00246071	0.04100834	-1.833236
LOC440359	-1.161555	11.2082995	-3.318116	0.00254774	0.04208736	-1.865822
TNFRSF17	0.96002186	6.83945223	3.31165096	0.00259009	0.04245763	-1.881275
B4GALT5	0.65492930	10.4320041	3.31002138	0.00260087	0.04250143	-1.885168
MED25	-0.889427	9.45365992	-3.309355	0.00260530	0.04253597	-1.88676
AHR	-0.73597	12.7422612	-3.302061	0.00265417	0.04317370	-1.904174
ARMET	0.70645941	9.33585446	3.29981358	0.00266940	0.04337640	-1.909535
KIR3DL2	-1.313526	8.42820838	-3.297905	0.00268240	0.04350985	-1.914087
NECAP2	-0.914476	10.4465843	-3.295503	0.00269886	0.04368148	-1.919816

LOC642103	0.75157273	8.19222953	3.28817061	0.00274968	0.04424330	-1.937289
BTN3A2	-0.789599	8.58360546	-3.287253	0.00275611	0.04428718	-1.939475
VNN2	0.68624927	12.7706348	3.27989923	0.00280813	0.04489684	-1.956981
IL8	-0.606817	7.02469619	-3.279345	0.00281209	0.04494110	-1.9583
C12ORF35	-0.618287	9.01475703	-3.271068	0.00287184	0.04566431	-1.977982
GIMAP5	-0.589442	9.53444838	-3.264631	0.00291917	0.04606778	-1.993276
DEFA3	1.99439063	10.8573502	3.26244382	0.00293541	0.04627229	-1.998469
LDHB	-0.794635	9.85738303	-3.261365	0.00294346	0.04633501	-2.00103
COL4A3BP	0.87404588	7.88389430	3.26088724	0.00294703	0.04637186	-2.002164
AKR1C3	-0.636969	7.26674684	-3.259326	0.00295872	0.04651711	-2.005868
ALS2CR2	-1.537974	11.8943571	-3.246569	0.00305598	0.04762992	-2.036118
BASP1	0.59558758	13.2850718	3.24402395	0.00307574	0.04789360	-2.042145
LRG1	0.81330711	10.0091687	3.23939587	0.00311200	0.04830379	-2.053102
HS.573503	-1.185319	7.72968203	-3.230728	0.00318101	0.04911275	-2.073606
ZDHHC19	1.77299506	7.0637895	3.22852609	0.00319878	0.04928632	-2.07881
ALPK1	0.62014636	8.37300465	3.22257620	0.00324726	0.04978966	-2.092866
STOML2	0.61568936	8.19119626	3.22198071	0.00325215	0.04982421	-2.094272
