# Supplementary figures and images for: Machine learning-driven discovery of NETs-associated diagnostic biomarkers and molecular subtypes in tuberculosis
Source: Front Cell Infect Microbiol. 2025 Oct 1;15:1591464. doi: 10.3389/fcimb.2025.1591464 (PMC12521227; doi:10.3389/fcimb.2025.1591464)

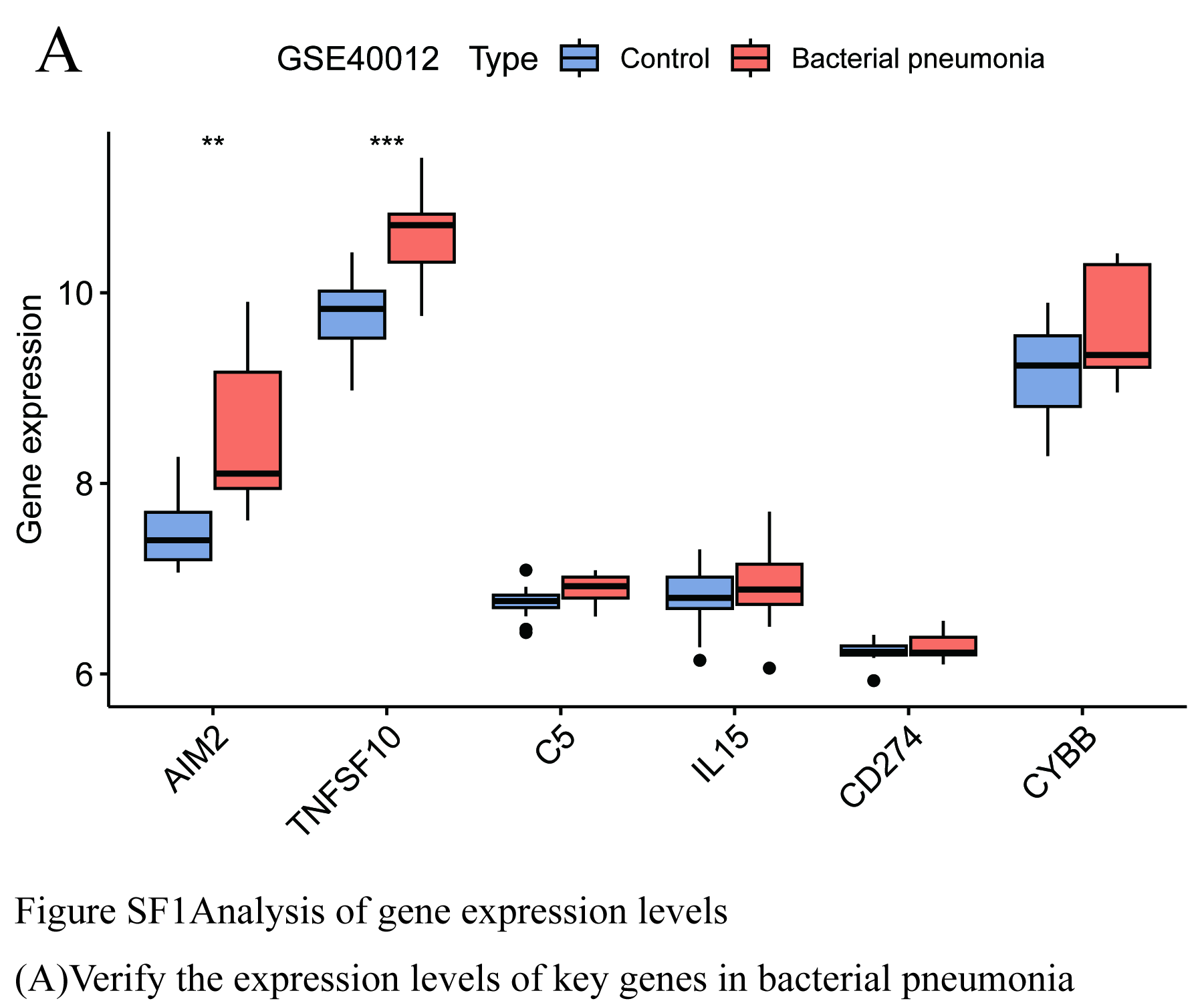

Supplement: Supplementary file 3 [file Image1.tif]
